# The bill of aging: fiscal projections of demographic changes on South Korea’s national health insurance, 2023–2042

**DOI:** 10.1186/s13561-025-00690-z

**Published:** 2025-11-17

**Authors:** Younhee Kim, Kyung-sook Woo

**Affiliations:** 1 Department of Social and Preventive Medicine, College of Medicine, Inha University, Incheon, South Korea; 2 Center for Health Economics & Policy Innovation, Imperial Business School, Imperial College London, London, UK; 3 Department of Preventive Medicine, Graduate School of Public Health, Hanyang University, Seoul, South Korea

## Abstract

**Background:**

Demographic shifts driven by declining birth rates and rising life expectancies pose significant challenges to healthcare systems globally, particularly in terms of financial sustainability. We projected fiscal trajectories for Korean National Health Insurance (NHI) by jointly forecasting revenues and expenditures for 2023–2042 and by quantifying the incremental fiscal effects of demographic change.

**Methods:**

The study employed a hybrid forecasting framework combining component-based projections with time-series econometric models. NHI revenues were projected by categorizing them into contributions, government subsidies, and other revenue. Expenditures were projected into medical benefits, non-medical benefits, and administrative costs. The impact of demographic changes was assessed by comparing the baseline scenario with a hypothetical scenario where the population structure remained constant at 2023 levels.

**Results:**

Projections indicate that NHI expenditures will surpass revenues starting in 2025, with revenues reaching 314.2 trillion KRW and expenditures 437.5 trillion KRW by 2042. Accumulated reserves are expected to be depleted by 2030, with annual deficits growing from 21.8 trillion KRW in 2032 to 123.3 trillion KRW in 2042. Compared to a scenario where population structure remains at 2023 levels, demographic shifts are projected to decrease revenues by 8.5 trillion KRW (4.5%) and increase expenditures by 30.8 trillion KRW (17.8%) in 2032, and by 42.7 trillion KRW (12.0%) and 109.7 trillion KRW (33.5%) respectively by 2042. Consequently, the annual fiscal burden attributable to demographic changes is estimated at 39.4 trillion KRW in 2032 (19.3% of total expenditures) and 152.5 trillion KRW in 2042 (34.8% of total expenditures).

**Conclusion:**

This study highlights the urgent need for policy reforms to address the “bill of aging” and ensure the long-term sustainability of South Korea’s NHI system. Policy responses must address this imbalance through integrated health expenditures monitoring, system efficiency improvements, sustainable financing including contribution rate reform and revenue diversification, community-based care infrastructure for aging populations, and maintained health equity. These findings offer valuable guidance for policymakers worldwide confronting the financial challenges of aging populations, emphasizing the need for comprehensive policy interventions to maintain financially sustainable healthcare systems for future generations.

**Supplementary Information:**

The online version contains supplementary material available at 10.1186/s13561-025-00690-z.

## Background

Demographic shifts, driven by declining birth rates and rising life expectancies, present significant challenges to healthcare systems globally [1]. These shifts reduce the working-age population, thereby diminishing the capacity to fund healthcare, while simultaneously increasing the demand for healthcare and long-term care services due to the growing elderly population. The financial sustainability of healthcare systems is increasingly challenged by these demographic changes, particularly those associated with accelerated population aging [1–7].

In response to these challenges, several fiscal projection reports, particularly those led by the Organization for Economic Co-operation and Development (OECD), have been published to assess healthcare system sustainability. These reports provide long-term forecasts of health spending, offering critical insights into future financial demands. For instance, the OECD’s 2024 Fiscal Sustainability Report projects that by 2040, average health spending across OECD countries, funded by public resources, will increase to between 11.2% and 11.8% of GDP, up from 8.8% in 2018, outpacing both the overall economy and government revenues [8]. Additionally, demographic factors, including population aging, account for just over a quarter of the projected growth in health spending across OECD countries [2]. Similarly, the 2021 Ageing Report provides a comprehensive analysis of the long-term fiscal impacts of demographic shifts across the European Union (EU) from 2019 to 2070. The report emphasizes the significant increase in the old-age dependency ratio, projected to rise from 34% in 2019 to 59% by 2070, which is expected to result in a marked increase in age-related public expenditures, particularly in pensions, healthcare, and long-term care [9].

Although numerous studies project health expenditures bcause of population aging, these studies have several limitations [2, 6, 9–14]. First, most studies predominantly focus on expenditure, often neglecting the revenue aspect of healthcare financing, which is crucial for a comprehensive assessment of sustainability. In addition, while many studies illustrate the increase in health expenditures due to demographic changes, they often fall short of examining how these demographic shifts affect the overall financial sustainability of healthcare systems.

South Korea presents a particularly compelling case study for examining these issues [15]. The country operates a universal health coverage system, ensuring that the entire population is covered either by the National Health Insurance (NHI) or the tax-based Medical Aid Program [16]. The NHI is a social insurance program that provides healthcare coverage to all citizens, funded by contributions from individuals, employers, and government subsidies [17]. Within this context, the challenges to the financial sustainability of South Korea’s healthcare system are particularly severe and urgent. The country is experiencing rapid aging due to one of the lowest fertility rates among OECD countries and a high life expectancy. The proportion of the population aged 65 and above is projected to increase from 14.3% in 2018 to 40.1% by 2050 [18]. Moreover, the old-age dependency ratio is expected to surge from 25.8 in 2023 to 77.3 by 2050, meaning that by 2050, each elderly person will be supported by just 1.3 working-age individuals, compared to four in 2023. These demographic shifts are significantly impacting the NHI system. As of 2022, NHI medical expenses for those aged 65 and older accounted for 43.2% of total NHI medical expenses [19].

In this context, the study has two aims. The first is to project both NHI revenues and expenditures for 2023–2042 and to identify the timing and size of deficits as well as the depletion of accumulated reserves. The second is to quantify the marginal impact of demographic change by comparing a baseline projection with a counterfactual in which the population structure is fixed at its 2023 composition. Accordingly, we examine four questions. First, we project the paths of NHI revenues and expenditures under baseline assumptions. Second, we identify when deficits arise and when reserves are depleted. Third, we quantify the portion of the fiscal trajectory attributable to demographic change by contrasting the baseline with the fixed-2023 population counterfactual. Fourth, we conduct sensitivity analyses across key parameters—including population projections, income and expenditure uncertainties, and contribution rate policies—and determine the contribution rates required to maintain annual fiscal balance.

The findings of this study are expected to contribute significantly to policy discussions and decision-making processes regarding the long-term sustainability of healthcare systems in rapidly aging societies. By providing a more holistic understanding of the financial implications of demographic shifts, this research aims to inform the development of effective strategies and structural reforms necessary to maintain the quality and accessibility of healthcare services in the face of demographic challenges.

## Methods

### Data sources

Demographic, economic, and NHI financial indicators were employed for the NHI financial projection. Population data were derived from the central population figures (as of July 1 each year) reported by Statistics Korea up to 2022 for actual figures, and from 2023 onwards, the medium scenario from population projections was utilized in the base analysis.

Economic indicators included GDP per capita with annual GDP growth rates and nominal wage growth rates, with actual values through 2024 obtained from Statistics Korea. Economic forecasts from 2025 onwards were based on long-term fiscal projections from the National Assembly Budget Office [20].

To evaluate the financial sustainability of the NHI, cash flow data provided by the National Health Insurance Service (NHIS) were utilized. To enhance projection accuracy, we obtained detailed statistics from NHIS on NHI contributions and medical benefits, the main sources of NHI revenue and expenditure. For NHI contributions, we obtained data on contributions per insured and the number of insured individuals by insured type (industrial workers and self-employed individuals) and age group (15–19, 20–24, …, 80–84, 85+) for 2003–2022. For medical benefits, we obtained data on medical benefits per capita and the number of healthcare users for both survivors and decedents by age group (0–4, 5–9, …, 95–99, 100+) for 2009–2023.

Basic statistics for demographic, economic, and health insurance financial indicators are presented in Table 1 and Supplementary Fig. 1.

**Table 1:** Demographic, economic, and NHI financial indicators for NHI financial projections in South Korea

	Demographic indicator		Economic indicator		NHI financial Indicators		
Year	Population [thousand people] (annual growth rate %)	Old-age dependency ratio	GDP per capita [thousand KRW] (annual growth rate %)	Nominal wage growth rate (%)	NHI revenue [trillion KRW] (annual growth rate %)	NHI expenditure [trillion KRW] (annual growth rate %)	NHI contribution rate [%] (annual growth rate %)
2010	49,554 (0.5)	14.8	27,837 (9.9)	2.2	34 (7.6)	35 (11.8)	5.33 (4.90)
2012	50,200 (0.5)	15.6	29,974 (3.9)	4.1	42 (10.1)	39 (3.8)	5.80 (2.80)
2014	50,747 (0.6)	16.8	32,288 (4.3)	3.2	49 (7.4)	44 (5.7)	5.99 (1.70)
2016	51,218 (0.4)	18.0	35,789 (5.3)	3.4	56 (6.3)	53 (9.1)	6.12 (0.82)
2018	51,585 (0.4)	19.6	38,906 (3.8)	4.6	62 (7.1)	62 (8.7)	6.24 (2.04)
2020	51,836 (0.1)	21.8	39,711 (0.9)	1.3	73 (7.9)	74 (4.1)	6.67 (3.20)
2022	51,673 (−0.2)	24.4	44,971 (4.6)	7.8	89 (10.3)	85 (9.6)	6.99 (1.89)
2024	51,751 (0.1) ^P^	27.4 ^P^	49,407 (6.2)	2.7	99 (4.4)	97 (7.2)	7.09 (0.00)
2026	51,609 (−0.1) ^P^	31.3 ^P^	53,668 (4.1)	3.6 ^P^			7.19 (1.48)
2028	51,460 (−0.1) ^P^	34.8 ^P^	58,216 (4.0) ^P^	3.7 ^P^			7.41 (1.48) ^P^
2030	51,306 (−0.2) ^P^	38.0 ^P^	63,058 (3.9) ^P^	3.8 ^P^			7.63 (1.48) ^P^
2032	51,135 (−0.2) ^P^	41.4 ^P^	68,142 (3.7) ^P^	3.8 ^P^			7.85 (1.48) ^P^
2034	50,938 (−0.2) ^P^	45.5 ^P^	73,448 (3.6) ^P^	3.8 ^P^			8.09 (1.48) ^P^
2036	50,701 (−0.2) ^P^	50.1 ^P^	78,985 (3.4) ^P^	3.9 ^P^			8.33 (1.48) ^P^
2038	50,417 (−0.3) ^P^	54.7 ^P^	84,759 (3.3) ^P^	3.9 ^P^			8.58 (1.48) ^P^
2040	50,059 (−0.4) ^P^	59.1 ^P^	90,810 (3.1) ^P^	3.9 ^P^			8.84 (1.48) ^P^
2042	49,625 (−0.5) ^P^	62.6 ^P^	97,258 (3.0) ^P^	3.9 ^P^			9.10 (1.48) ^P^

^P^, projected; *NHI*, National Health Insurance; 1USD = 1,382 KRW (as of July 31, 2025); Data are presented at two-year intervals; Old-age dependency ratio is defined as the population aged 65 and over per 100 persons aged 15-64; The NHI contribution rate is assumed to grow annually by 1.48% starting in 2026, reflecting the most recent rate increase trend; Values in parentheses indicate annual growth rates

Source: Demographic indicators - actual values and projections from Statistics Korea; Economic indicators - actual values from Statistics Korea, projections from the National Assembly Budget Office (2025a); NHI financial indicators - actual values from the National Health Insurance Service (NHIS).

### Financial projections method

**Fig. 1:**
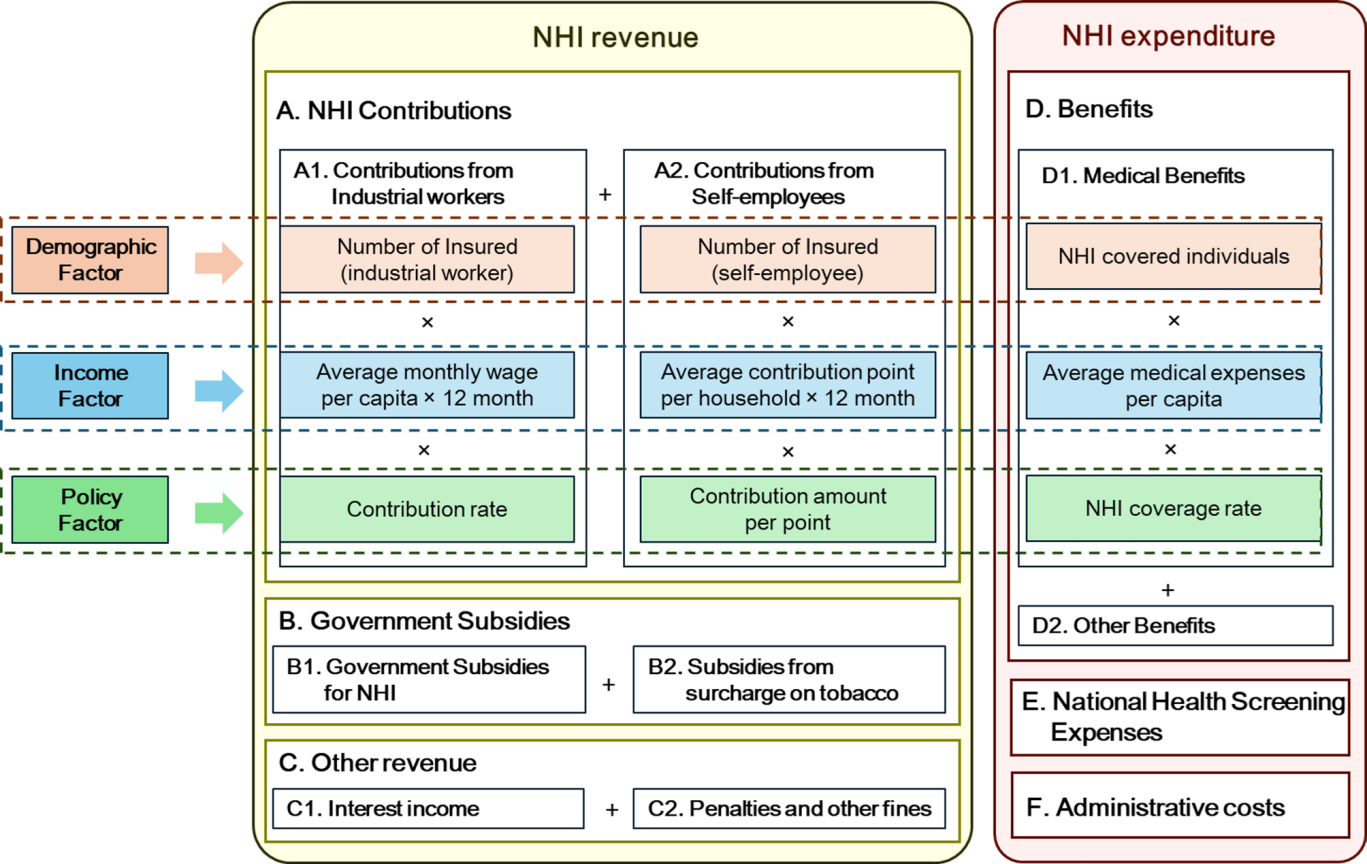
Overall structure and projection methods for Korean national health insurance finances Note: NHI, National Health Insurance

This study projects the NHI finances from the perspective of the National Health Insurance Service (NHIS), the single-payer of Korea's NHI. The overall structure and projection methodology for NHI finances are illustrated in Fig. 1. The projections incorporate actual financial data through 2024, as aggregate statistics for 2024 were recently published.

1. NHI Revenue
  NHI revenue was projected by categorizing it into NHI contributions (A), government subsidies (B), and other revenue (C). Although NHIS operates as a single-payer, the NHI contribution calculations differ between industrial workers and self-employed individuals. Therefore, the contributions from industrial workers (A1) and self-employed (A2) were projected separately. NHI contributions (A) were projected by estimating the number of insured individuals based on their proportion to the population and the annual average contribution per insured person in each age group (15–19, 20–24, 25–29, …, 75+). For industrial workers (A1), the annual average contribution per insured was calculated by multiplying the average per capita wage by the contribution rate. NHI contributions from industrial workers are shared equally between employees and employers, with each party bearing 50% of the total contribution burden. For self-employed individuals (A2), the annual average contribution was calculated by multiplying contribution points per household by the monetary value per scoring point. Given the ongoing NHI contribution system reform implemented between 2018 and 2022, we applied the growth rate observed prior to the reform period rather than the recent post-reform trends. Specifically, the average growth rate from 2018 to 2021 was applied to the current contribution point levels, as the growth patterns immediately following the major reforms were considered unlikely to persist in the long-term. The NHI contribution rate is assumed to grow annually by 1.48% starting in 2026, reflecting the most recent rate increase trend. Under this baseline scenario, the contribution rate would increase from 7.19% in 2026 to 7.85% in 2032 and 9.10% in 2042, approaching the 8% statutory ceiling by 2034.
  Government subsidies (B) were projected by separating them into subsidies from the national general account (B1) and those from the tobacco surcharge (B2). For government subsidies from the general account, the 2025 budgeted amount was applied for that year, while projections from 2026 onwards were calculated by applying the average proportion of government subsidies relative to NHI contributions observed in 2024 (12.2%) to projected contribution revenue. Subsidies from the tobacco surcharge were assumed to remain at the 2025 budget level throughout the projection period. This assumption is based on two considerations. First, under current regulations, only up to 65% of tobacco surcharge revenue can be allocated to NHI financing. Second, given declining smoking rates and stabilizing tobacco consumption patterns, continued growth in tobacco surcharge revenue appears unlikely.
  Other revenue (C) included interest income (C1) and penalties and other fines (C2). Interest income was calculated by multiplying the projected interest rate by the previous year’s accumulated reserves. Other income excluding interest income was estimated by applying the average proportion relative to NHI contributions observed in recent years (2.5% based on 2024 data).

(2) NHI Expenditure
  Health insurance expenditures were projected in three categories: medical benefits (D), non-medical benefits (E), and administrative costs (F). Medical benefits (D) were calculated by multiplying the number of NHI-covered individuals in each age group, separated into survivors and decedents, by the annual average medical expense per capita and the NHI coverage rate. The number of NHI-covered individuals in each age group (0–4, 5–9, …, 100+) was estimated by applying the proportion of NHI-covered individuals relative to the total population in the most recent year (2023) to the population projection for each age group. The number of decedents was calculated by multiplying the number of NHI-covered individuals in each age group at the end of the previous year by age-specific mortality probabilities.
  Non-medical benefits (E) comprise national health screening expenses (E1) and cash benefits (E2). National health screening expenses include general health screening, cancer screening, dental screening, and health screenings for infants. These expenses were estimated by multiplying the proportion of screening participants relative to the population by the average screening cost per capita, both estimated for each projection year. Age-specific participation rates were applied to reflect demographic changes in the population structure, while per capita screening costs were differentiated between infant and adult populations. The average annual growth rates over the most recent five years were applied to project per capita screening costs for both age categories. Cash benefits (E2), which include maternity and childbirth support and reimbursements for out-of-pocket expenses exceeding the catastrophic coverage threshold, were assumed to increase proportionally with medical benefits.
  Administrative costs (F) were estimated by applying the ratio of administrative costs to NHI benefit expenditures to the projected benefit amounts. Given the declining trend in this ratio over recent years, the projection incorporated this observed decreasing pattern to reflect ongoing efficiency improvements in administrative operations.

### Projection models

This study employed a hybrid forecasting framework combining component-based projections with time-series econometric models [10]. ARIMAX models were utilized to estimate key variables within the component framework, specifically average monthly wages per capita for industrial workers (revenue component) and average medical expenses per capita (expenditure component).

ARIMA modeling captures dynamic patterns within time series through autoregressive, differencing, and moving average parameters [21–23]. The extension to ARIMAX models incorporates exogenous variables, improving forecast accuracy when external factors significantly influence the dependent variable [24]. ARIMAX models can handle both internal time series patterns and external economic influences, making them suitable for health expenditure forecasting. In our analysis, we incorporated GDP per capita, nominal wages, and COVID-19 impact indicators to capture macroeconomic effects and pandemic disruptions on NHI finances.

As an initial step in model specification, stationarity was assessed using the Augmented Dickey-Fuller test for all income and expense series [21–25]. Results indicated that most series violated the stationarity assumption, necessitating first-differencing (d = 1) to achieve stationarity. The survivors’ 5–9 age group expense series exhibited stationarity, thus both differenced and non-differenced specifications were evaluated as candidate models to ensure optimal model selection.

For series requiring differencing, a restricted set of non-seasonal ARIMAX (p, d, q) candidates was evaluated, where p is the autoregressive order, d is the differencing order, and q is the moving average order. These candidates included ARIMAX(1,1,1), ARIMAX(0,1,1), ARIMAX(1,1,0), and ARIMAX(0,1,0) specifications. Expense series for younger age groups (0–4, 5–9, 10–14, and 15–19 years cohorts) exhibited excessive volatility unsuitable for ARIMAX modeling; for these series, we adopted historical trend extrapolation.

For income projections, ARIMAX models incorporated nominal wages as exogenous variables, which demonstrated better BIC values compared to per-capita GDP specifications. Medical expense projections utilized ARIMAX specifications that included per-capita GDP as an economic driver alongside COVID-19 pandemic dummy variables. Seven different pandemic impact scenarios were tested: individual year effects (2020, 2021, 2022), multi-year period effects (2020–2021, 2021–2022, 2020–2022), and no pandemic impact. For survivors aged 25–29, an additional specification incorporating effects for years 2020 and 2022 was evaluated based on observed trend patterns in the data.

Model selection was based on the Bayesian Information Criterion (BIC) across all combinations of ARIMAX specifications and COVID-19 variables, with additional requirements for convergence and parameter stability. Models with convergence failures were excluded despite having lower BIC values, prioritizing model reliability.

Selected models underwent residual diagnostics including examination of residual autocorrelation through ACF and PACF plots, application of the Ljung-Box Q test for serial correlation, and assessment of residual normality using histogram distributions and Q-Q plots. The diagnostic results for the final selected models indicated no substantial violations of model assumptions.

Based on the selected model specifications and estimated parameters, projections for age-specific income and medical expense series were generated through 2042.

The final model orders, differencing parameters, and pandemic dummy specifications for each time series are documented in the supplementary materials (Supplementary Tables 1–4).

### Sensitivity analyses 

To assess the robustness of baseline projections and quantify uncertainty, sensitivity analyses were conducted on key parameters affecting NHI finances.

First, alternative population scenarios were examined using Statistics Korea’s high, medium, and low projection scenarios. Corresponding macroeconomic projections for each demographic scenario, provided by the National Assembly Budget Office, were applied to capture the effects on both revenues and expenditures.

Second, uncertainty in income and expenditure projections was assessed by applying confidence intervals to age-specific parameters. For variables estimated using ARIMA models (age-specific income per insured and medical expenses per capita for survivors and decedents), 95% confidence interval bounds were applied. For parameters estimated using historical growth rates (income for industrial workers aged 75+, contribution points for self-employed, and medical expenses for ages 0–19), ± 3% variations were applied to reflect estimation uncertainty.

Third, Sensitivity analysis examined different contribution rate growth scenarios to assess revenue impacts. Annual growth rates of 1%, 1.48% (baseline), and 3% were applied to the contribution rate, both with and without the 8% statutory ceiling specified in the National Health Insurance Act.

Fourth, we examined scenarios where medical expense growth for those aged 65 and older moderates due to accelerated healthy aging or technology-driven efficiency improvements (e.g., AI, telemedicine). Per capita medical expenses for this age group were modeled as reduced by 3% and 5% relative to baseline levels each year.

Fifth, given NHI’s requirement to maintain annual fiscal balance, we simulated the contribution rates necessary to achieve fiscal balance in each projection year. Starting from 2027 (as 2026 rates are predetermined), we iteratively calculated the minimum contribution rate required to ensure annual revenues equal or exceed expenditures, thereby preventing fiscal deficits.

## Results

### Time series modeling and projection parameters

ARIMAX models were successfully fitted to income and medical expense series across all age groups. Stationarity tests indicated that most series required first-differencing (d = 1), with only the survivors’ 5–9 age group exhibiting stationarity in levels (Supplementary Table 1). Model selection based on BIC, convergence, and stability criteria identified optimal specifications for each series. Income projections predominantly employed ARIMAX(0,1,0) specifications with nominal wages as exogenous variables. Medical expense series demonstrated greater complexity, with ARIMAX(0,1,1) and ARIMAX(1,1,0) specifications most commonly selected. COVID-19 pandemic effects varied substantially across age groups, with multi-year impact periods (2020–2022) frequently selected for older cohorts. Due to high volatility, trend extrapolation was applied for the youngest age groups (0–19 years) in survivor and decedent expenses. Detailed model specifications, BIC comparisons, and diagnostic results are presented in Supplementary Tables 2–4.

### Population and insurance coverage projections

According to population projections, the number of insured industrial workers (excluding dependents) is expected to increase until 2033 and then decrease, while the number of insured self-employed (based on household heads) is projected to continue increasing, although at a slower rate, until 2042 (Supplementary Fig. 2). The number of insured industrial workers in NHI is estimated to rise from 20.08 million in 2023 (38.8% of the total population) to 23.35 million in 2032 (45.7% of the total population), averaging a 1.7% annual increase before declining to 21.00 million in 2042 (42.3% of the total population). The number of self-employed contributors is anticipated to increase from 7.96 million in 2023 (15.4% of the total population) to 10.48 million in 2032 (20.5% of the total population) at an average rate of 3.1% annually, with a slowdown thereafter, reaching 13.09 million in 2042 (26.4% of the total population). The total number of NHI insured is expected to peak at 34.08 million in 2034 and then continuously decline.

### NHI financial projections and fiscal impact

**Fig. 2:**
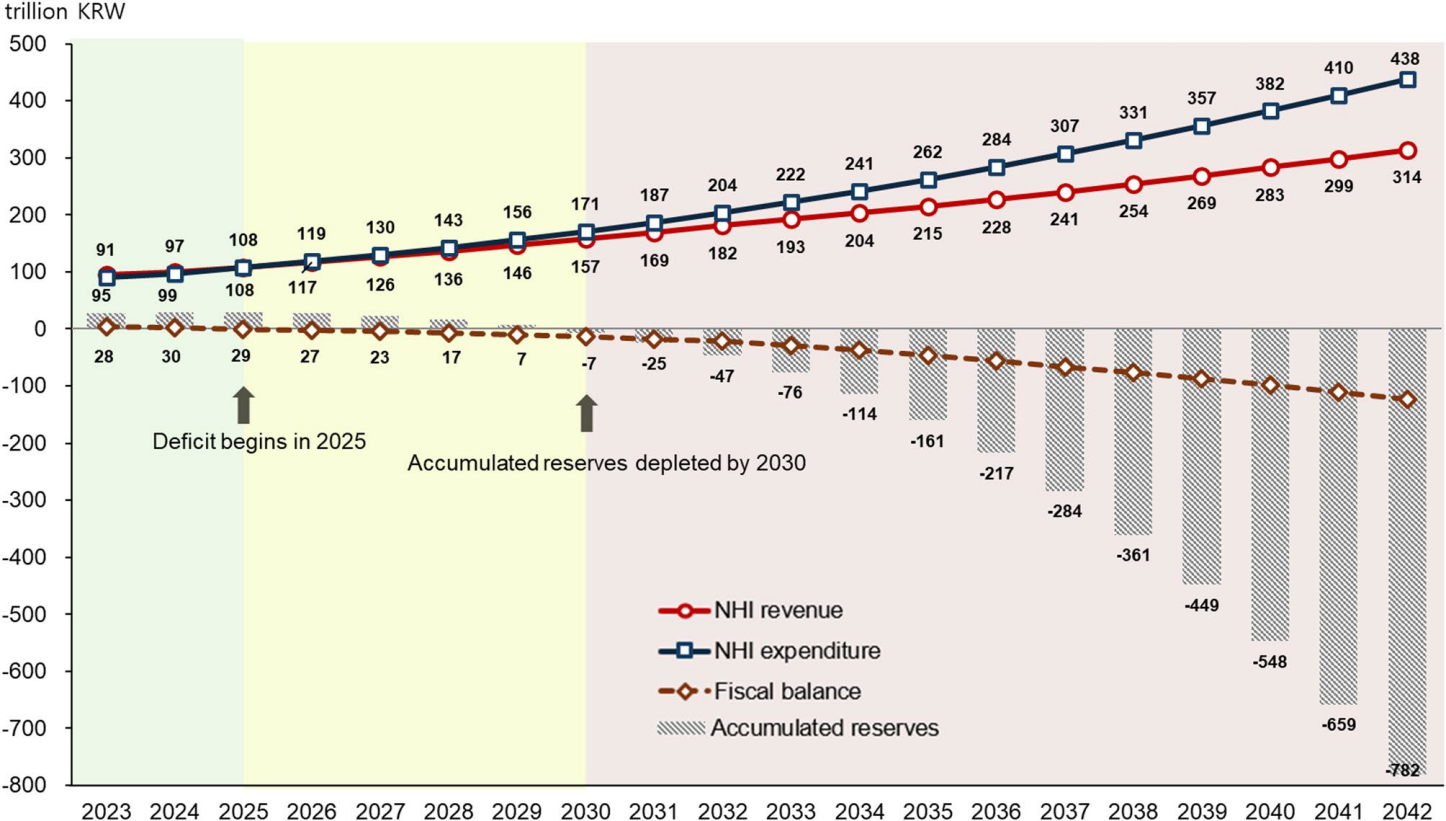
NHI financial projections: baseline scenario Note: NHI, National Health Insurance; KRW, Korean Won; 1USD = 1,382 KRW (as of July 31, 2025)

Projections of NHI finances, incorporating historical trends such as contribution rate increases and medical expense growth, indicate a deficit beginning in 2025, with reserve depletion by 2030 and annual deficits reaching 21.8 trillion KRW in 2032 and 123.3 trillion KRW in 2042 (Fig. 2). Assuming an average annual contribution rate increase of 1.48% (2026 recent increase rate) without an 8% ceiling, NHI revenues are projected to be 108.0 trillion KRW in 2025, 181.8 trillion KRW in 2032, and 314.2 trillion KRW in 2042. The average annual revenue growth rate is expected to decrease from 7.5% in 2023–2032 to 5.6% in 2033–2042. NHI expenditures are projected to be 108.4 trillion KRW in 2025, 203.7 trillion KRW in 2032, and 437.5 trillion KRW in 2042, with average annual growth rates of 9.4% in 2023–2032 and 7.8% in 2033–2042.

The share of total medical benefits for those aged 65 and older is projected to rise from 45.1% in 2023 to 57.6% in 2032 and 66.4% in 2042, reflecting average annual growth rates of 12.6% and 9.4% respectively, compared to 6.5% and 5.4% for those under 65 (Supplementary Fig. 3). This concentration of expenditures among older populations is further evident in per capita medical expenses, which demonstrate substantial and widening age gradients (Supplementary Fig. 4). In 2023, average per capita medical benefits for those aged 65 and older (4.0 million KRW) were 5.6 times higher than for those aged 20–39 (0.7 million KRW), widening to 5.9 times in 2032 (7.9 vs. 1.3 million KRW) and 6.5 times in 2042 (15.5 vs. 2.4 million KRW), reflecting faster annual growth rates among older adults (7.4%) compared to younger cohorts (6.4%).

### Demographic impact analysis

**Fig. 3:**
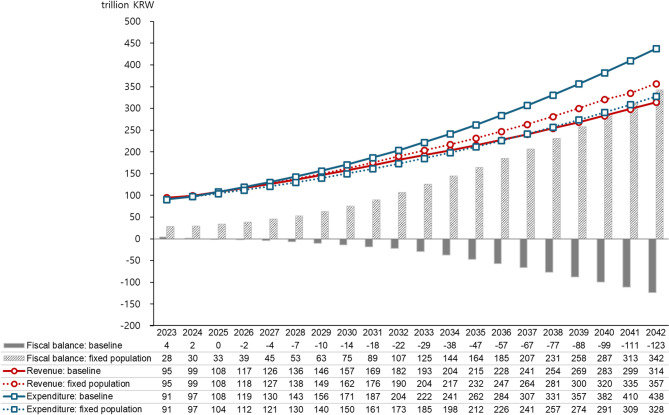
Impact of demographic changes on NHI financial projections: baseline scenario vs. fixed population *KRW,* Korean Won; 1USD = 1,382 KRW (as of July 31, 2025)

According to Statistics Korea’s population projections, the population is expected to decrease from 51.71 million in 2023 to 49.63 million in 2042. During this period, the proportion of the population aged 65 and over is projected to increase from 18.2% to 35.5%, with the old-age dependency ratio rising from 24.4 to 62.6. To assess the impact of these demographic changes on NHI finances, a comparison was made between the baseline scenario and a hypothetical scenario where the population structure and size remain constant at 2023 levels (Fig. 3).

The results show that in the baseline scenario, compared to the fixed population scenario, NHI revenues in 2032 are projected to be 8.5 trillion KRW (4.5%) lower, while expenditures are expected to be 30.8 trillion KRW (17.8%) higher. By 2042, revenues are projected to be 42.7 trillion KRW (12.0%) lower, and expenditures 109.7 trillion KRW (33.5%) higher. Consequently, the annual fiscal burden due to demographic changes is estimated to increase by 39.4 trillion KRW in 2032 (19.3% of 2032 expenditures) and 152.5 trillion KRW in 2042 (34.8% of 2042 expenditures). If the population structure were to remain at current levels, the fiscal balance is projected to maintain a surplus, even with continued growth in per capita medical expenses.

### Sensitivity analyses

Sensitivity analyses revealed some differences in fiscal outcomes across parameter scenarios, highlighting key sources of uncertainty in long-term projections (Supplementary Tables 5–9 & Supplementary Figs. 5–10).

Population scenarios demonstrated modest impact on fiscal balance. Under the high population scenario, both revenues and expenditures increased relative to baseline, while the low population scenario reduced both components. These offsetting changes resulted in minimal net effects on fiscal balance (Supplementary Tables 5 & Supplementary Fig. 5).

Income projection uncertainty affected fiscal sustainability timelines. Using the upper 95% confidence bound for income projections delayed deficit onset from 2025 to 2029 and postponed reserve depletion from 2030 to 2033. Similarly, applying the lower 95% CI for expenditure projections shifted deficit onset to 2029 and reserve depletion to 2033 (Supplementary Tables 6–7 & Supplementary Figs. 6–7).

Technology-diffusion and healthy aging scenarios with reduced medical expense growth for those aged 65 and older delayed deficit onset by 1–2 years and reserve depletion by 1–2 years. The 3% reduction scenario postponed deficit onset to 2026 and reserve depletion to 2031; the 5% scenario delayed them to 2027 and 2032, respectively (Supplementary Tables 8 & Supplementary Fig. 8).

Contribution rate policies showed strong influence on fiscal trajectories (Supplementary Tables 9 & Supplementary Fig. 9). Under the baseline 1.48% annual growth rate with the 8% statutory ceiling, contribution rates would reach the ceiling by 2034, constraining revenue growth thereafter. Reducing the annual growth to 1% accelerated fiscal deterioration with larger deficits and earlier reserve depletion. A 3% annual growth improved fiscal outcomes, postponing reserve depletion by three years to 2033, though deficits still emerged.

Break-even contribution rate simulations revealed substantial adjustments required to maintain annual fiscal balance (Supplementary Fig. 10). The contribution rate would need to reach 7.55% in 2027 (4.9% annual growth rate) and continue rising at approximately 3% annually thereafter, reaching 12.71% by 2042.

## Discussion

This study presents financial projections for South Korea’s NHI), examining the impacts of demographic shifts on healthcare financing sustainability. The projections indicate that NHI expenditures will surpass revenues starting in 2025, leading to financial deficits, with accumulated reserves expected to be depleted by 2030. Annual deficits are projected to reach 21.8 trillion KRW in 2032 and 123.3 trillion KRW in 2042.

Sensitivity analyses revealed some uncertainty in these projections. More favorable assumptions for income projections for industrial workers and contribution points for self-employed individuals, as well as lower medical expense estimates, or technology-driven cost reductions for older populations (3–5% annual savings) could delay the timing of deficits and reserve depletion by approximately one to four years. However, despite this uncertainty, fiscal imbalances remain inevitable beyond the medium term, with deficits emerging consistently across all sensitivity scenarios after 2030.

Demographic changes play a substantial role in these fiscal pressures. The primary driver of these fiscal challenges is the shift in South Korean population structure. The old-age dependency ratio is projected to rise from 25.8 in 2023 to 62.6 by 2042, meaning each elderly person will be supported by 1.6 working-age individuals, compared to four in 2023. This demographic transition drives NHI expenditures upward, with those aged 65 and older accounting for an increasing share of total medical benefits. Their share rose from 45.1% in 2023 and is projected to reach 57.6% in 2032 and 66.4% in 2042. Compared to a scenario where population structure remains at 2023 levels, demographic shifts are projected to decrease revenues by 8.5 trillion KRW (4.5%) and increase expenditures by 30.8 trillion KRW (17.8%) in 2032, and by 42.7 trillion KRW (12.0%) and 109.7 trillion KRW (33.5%) respectively by 2042. Consequently, the annual fiscal burden attributable to demographic changes is estimated at 39.4 trillion KRW in 2032 (19.3% of total expenditures) and 152.5 trillion KRW in 2042 (34.8% of total expenditures). If population structure were to remain constant, the NHI would maintain fiscal surplus throughout the projection period despite continued growth in per capita medical expenses.

Population projections are fundamentally determined by three components: fertility rates, mortality rates, and net international migration. Historically, South Korea’s population trajectories have been predominantly shaped by declining fertility and declining mortality, with international migration playing a relatively minor role. South Korea’s net international migration has historically been modest, approximately 65 thousand persons annually as of 2025, representing less than 0.13% of the total population. Statistics Korea’s population projections assume relatively stable migration patterns based on these historical trends. However, confronting unprecedented demographic pressures driven by low fertility rates and rapid population aging, Korea may need to consider substantial immigration policy shifts [26, 27]. Such changes are partially reflected in Statistics Korea’s high population scenario, which incorporates moderately higher net migration assumptions. Nevertheless, major immigration policy reforms that substantially increase working-age migration were not modeled in this study. Such reforms would produce demographic and fiscal effects beyond our baseline and high-variant scenarios, potentially moderating both the decline in the working-age population and the rise in the old-age dependency ratio more substantially than currently projected.

Beyond demographic composition, labor market dynamics such as labor force participation and productivity growth may also affect NHI fiscal projections. Labor force participation was incorporated in our revenue projections through age-specific estimation of insured industrial workers based on historical trends. These participation rates have been gradually increasing over time and are assumed to continue this upward trend throughout the projection period. However, if labor force participation rates were to remain constant at current levels rather than continuing their historical growth, NHI contribution revenues would be lower than our baseline projections. Productivity improvements present more multifaceted effects, as they simultaneously influence both sides of the fiscal equation. Enhanced labor productivity primarily affects wages (and thus NHI contributions) and GDP per capita (which drives medical expense growth in our ARIMAX models). While higher productivity would increase contribution revenues, it could also elevate healthcare expenditures through greater purchasing power and demand for higher-quality medical services. The net fiscal impact therefore depends on the relative magnitude of these offsetting effects and remains context-dependent.

Technological innovation presents complex fiscal trade-offs. Historically, innovation has increased healthcare spending by introducing new treatments and capabilities [28–31]. However, emerging digital health technologies such as AI and telehealth may reduce costs through improved efficiency [32–36]. These cost-reduction effects may be particularly pronounced among older populations by reducing high-cost inpatient care, enabling home-based care alternatives, and enhancing patient-centered and supportive care delivery [32, 37, 38]. Based on this potential, we conducted sensitivity analyses assuming 3–5% slower annual medical expense growth for those aged 65 and older. If similar cost-moderating effects extended across all age groups, deficit onset would be further delayed, though fiscal imbalances would remain inevitable.

Compared with previous projections of Korean NHI expenditure, earlier studies estimated spending for 2040 between 275 and 416 trillion KRW, consistent with our recent estimate of 398.4 trillion KRW [39]. The OECD’s forecasts, which reflected trends up to 2018 and accounted for population aging, projected Korea’s health spending ratio to reach 9.7% of GDP by 2030. However, Korea had already reached this level by 2022, indicating more rapid growth than the OECD projected. A recent study, using updated data, projects medical spending to rise to about 12% of GDP by 2030, 15% by 2040, and nearly 20% by 2060 [14]. This trend shows health spending outpacing GDP growth, mainly due to rising healthcare demand and costs due to demographic changes in Korea.

The specification of COVID-19 impact periods introduced uncertainty in projection outcomes, with model forecasts showing sensitivity to assumptions about pandemic duration and timing. This study incorporated all available data while explicitly modeling COVID-19 effects through age-specific dummy variables, whereas some health financing projections exclude pandemic years to minimize data distortions [14, 39]. The impact of COVID-19 varied across age groups, reflecting Korean healthcare utilization patterns. Korean healthcare system is characterized by high utilization rates compared to OECD averages [40]. During the pandemic, younger age cohorts with historically lower healthcare utilization experienced increases in medical expenses, primarily due to COVID-19 testing and treatment. Conversely, older populations with traditionally high baseline utilization demonstrated reductions in healthcare use due to infection avoidance behaviors.

This pattern differs from other healthcare systems with different baseline accessibility. In the United States, Medicaid programs experienced expense increases during 2020–2022, driven primarily by eligibility expansions for previously uninsured populations, while private insurance systems showed more modest growth as utilization avoidance offset pandemic-related increases [6]. Given the distinct healthcare utilization patterns across age groups, the assumption of uniform COVID-19 impact across all ages would introduce bias. The variance in forecasting outcomes across different pandemic scenario specifications underscores the need for further research as additional post-pandemic data becomes available. Although our approach examines alternative specifications of pandemic-affected period, the accuracy of long-term projections would benefit from additional post-2022 observational data and more rigorous econometric models specifically designed to capture pandemic effects on healthcare utilization patterns.

In numerous projections of health expenditures influenced by population aging, the concept of “healthy aging” has been adopted [2, 8, 41]. Healthy aging suggests that the morbidity period remains constant even as longevity increases, potentially slowing the rate of health expenditure growth. However, this study does not separately incorporate assumptions related to healthy aging. Instead, the projections are based on the continuation of past trends, under the assumption that any effects of healthy aging are already reflected in historical data. Since historical expenditures already incorporate improvements in life expectancy and gradual health improvements, these factors are inherently embedded in the trends analyzed. Additionally, this study separates medical expenses into those for survivors and decedents, enabling the reflection of increasing medical expenses prior to death through changes in mortality probabilities. This approach allows for a more accurate representation of end-of-life medical expenses in the projection model.

Another consideration is the concept of income elasticity of demand, which has been discussed in previous research on health expenditure projections [2, 8, 9, 11]. Income elasticity, particularly in models that explain per capita health expenditures using per capita GDP, reflects the responsiveness of health spending to changes in income. It is generally anticipated that health expenditure will not increase as rapidly as income once certain thresholds are reached. However, this study did not explicitly incorporate income elasticity or employ log-transformed models for per capita health expenditure and GDP [42, 43]. This methodological choice reflects the focus of this study on projecting future values based on historical trends for detailed age groups rather than estimating elasticity parameters. Nevertheless, per capita GDP may serve as a composite indicator that captures much of the combined effect of multiple factors influencing NHI revenues and expenditures, including technological advancements and changing healthcare utilization patterns.

Policy recommendations to address these challenges should consider that NHI revenues are expected to grow more slowly than expenditures due to population aging, declining birth rates, and a low-growth economic environment. This fundamental imbalance between revenue growth and expenditure increases necessitates strategic policy interventions across multiple domains [8, 44–46]. First, comprehensive monitoring of health expenditures across all programs is essential. This includes integrating NHI and medical aid programs and examining interactions with long-term care insurance [45]. While interactions between NHI and private health insurance impact total healthcare spending, a comprehensive inter-system management plan has yet to be established [47]. Second, improving expenditure efficiency is vital [3, 16]. Sensitivity analyses demonstrated that reducing the average annual expenditure growth rate from 9.4% to 8.8% in 2023–2032 could delay both deficit onset and reserve depletion. For instance, in the OECD’s projection of health spending as a share of GDP, South Korea showed a significant difference between scenarios with and without effective cost containment policies [2]. Measures such as expanding primary care services, reassessing the use of medical technologies and pharmaceuticals with low cost-effectiveness, and promoting hospice and palliative care could support more sustainable service utilization. Emphasizing preventive care and functional capacity preservation for older people may help reduce costly acute care utilization in the long-term [44]. Third, securing sustainable public financial resources is essential. Given limitations in contributor growth, raising the NHI contribution rate appears inevitable. Sensitivity analyses showed that increasing the annual contribution rate growth from 1.48% to 3% could delay reserve depletion by three years, though deficits would still emerge. Furthermore, break-even simulations reveal that maintaining fiscal balance would require a 4.9% rate increase in 2027 followed by approximately 3–4% annual growth thereafter, ultimately reaching 12.71% by 2042. Given that this trajectory far exceeds the 8% statutory ceiling, legislative amendment of the contribution rate cap becomes essential for long-term fiscal sustainability. Explicitly defining government subsidies for NHI by law would ensure stable and predictable financing. Introducing health taxes on unhealthy foods such as those high in sugar or sodium could provide supplementary revenue [3, 48–50]. Fourth, responding to rapid population aging requires developing age-appropriate service delivery models and long-term care systems. Investing in community-based care infrastructure, workforce training for geriatric services, and assistive technologies can help meet the growing demand from older populations while reducing institutional care costs [44]. Lastly, while addressing financial sustainability, it is crucial to promote access for socially vulnerable populations [16]. The primary objectives of healthcare systems—income protection and guaranteed access—must be maintained. Efforts to manage expenditure should be accompanied by improvements in coverage for under-protected areas, reducing unmet needs, and mitigating catastrophic costs, thereby ensuring equity in healthcare access. This is particularly important for older adults, who face higher health risks and often have limited financial resources [51].

Several methodological limitations should be acknowledged. First, our ARIMAX models projected medical expenses for each age group over periods extending beyond the available historical observation periods, introducing uncertainty in long-term forecasts. Despite this uncertainty, sensitivity analyses demonstrated that fiscal imbalances remained consistent across parameter variations, with deficits emerging reliably after 2030 regardless of assumption changes.

Second, our models did not capture several important exogenous factors that could substantially alter NHI fiscal projections. Labor market dynamics, including productivity growth, were not explicitly incorporated despite their potential to affect both wages and medical expense patterns [52]. While our sensitivity analyses incorporated Statistics Korea’s high, medium, and low population scenarios—which reflect varying assumptions about fertility, mortality, and migration—substantial immigration policy shifts substantially beyond historical patterns were not modeled. Such reforms, potentially bringing substantially higher levels of working-age migration, could meaningfully alter both the dependency ratio and contribution base. Healthcare workforce constraints, particularly physician supply limitations that may not keep pace with aging-related demand increases, represented critical supply-side factors not reflected in our expenditure projections [14]. Third, while our projections incorporated historical trends that implicitly reflected gradual technological progress, these models did not account for potential rapid technological advances such as AI-enabled diagnostic support or digital health innovations that could fundamentally alter cost structures and utilization patterns [33, 34, 53]. Finally, although COVID-19 impacts were incorporated through age-specific adjustments, post-pandemic structural changes in healthcare utilization patterns may not have been fully captured given the limited post-pandemic observation period.

Despite these limitations, this study makes several important contributions. By jointly projecting both revenues and expenditures—an approach often neglected in health financing studies—it provides a comprehensive assessment of NHI fiscal sustainability. The methodology advances NHI finance projection by incorporating ARIMAX models within a component-based framework, enabling detailed age-specific projections that can inform policy decisions. The findings highlight critical financial pressures from demographic changes, providing evidence for prioritizing fiscal sustainability policies. The sensitivity analyses demonstrate that parameter uncertainty affects the timing but not the inevitability of fiscal challenges, while break-even simulations reveal the magnitude of contribution rate adjustments needed for sustainability. This methodological framework offers a valuable benchmark for international comparative studies of healthcare financing systems facing similar demographic transitions, contributing to broader understanding of sustainable public finance in aging societies.

## Conclusion

This study examines the fiscal challenges that South Korean NHI system will face due to demographic transitions. The projections show that expenditures will surpass revenues in the near term, with accumulated reserves depleted within the decade. Demographic changes account for a substantial and growing portion of fiscal pressures: the annual burden attributable to population aging is projected at 39.4 trillion KRW in 2032 (19.3% of total expenditures) and 152.5 trillion KRW in 2042 (34.8% of total expenditures), representing the incremental cost of an aging society compared to a scenario with constant 2023 population structure.

Given these demographic pressures, policy action is needed to address the “bill of aging.” The sensitivity analyses demonstrate that improvements in expenditure efficiency can delay fiscal deterioration. Priority actions include improving expenditure management through expanded primary care and reassessment of low-value medical technologies. Maintaining fiscal balance would require contribution rates to reach 12.71% by 2042, necessitating legislative amendment of the current 8% statutory ceiling. Diversifying revenue sources through introducing health taxes on unhealthy foods, such as sugar-sweetened beverages and high-sodium products could provide supplementary funding.

The “bill of aging” extends beyond South Korea. Many countries face similar demographic transitions that threaten healthcare system sustainability. The findings presented here offer guidance for policymakers worldwide confronting the financial challenges of aging populations. By prioritizing expenditure management and revenue diversification, South Korea and other countries facing similar demographic transitions can maintain financially sustainable healthcare systems that preserve quality and equitable access for future generations.

## Supplementary Information

Supplementary Material 1.

Supplementary Material 2.

## Data Availability

The demographic projections and economic indicators used in this study are derived from publicly available statistical data and reports. These can be accessed through the official websites of Statistics Korea (http:/kostat.go.kr) and the National Assembly Budget Office (https:/www.nabo.go.kr).The detailed health insurance statistics by specific categories, provided by the National Health Insurance Service (NHIS), were used under license for the current study and are not publicly available due to data protection regulations. However, aggregated data are available from the corresponding author upon reasonable request and with permission from the NHIS.
