# Supplementary material for: The bill of aging: fiscal projections of demographic changes on South Korea’s national health insurance, 2023–2042
Source: Health Econ Rev. 2025 Nov 17;15:97. doi: 10.1186/s13561-025-00690-z (PMC12625416; doi:10.1186/s13561-025-00690-z)
Supplement: Supplementary file 1 — Supplementary Material 1. [file 13561_2025_690_MOESM1_ESM.docx]

**Supplementary Materials 1**

**List of Supplementary Tables**

Supplementary Table 1. Stationarity test: Augmented Dickey-Fuller (ADF) test statistics 2

Supplementary Table 2. ARIMAX model fit comparison (BIC) and model selection: income 4

Supplementary Table 3. ARIMAX model fit comparison (BIC) and model selection: expenditure of survivors 5

Supplementary Table 4. ARIMAX model fit comparison (BIC) and model selection: expenditure of decedents 9

Supplementary Table 5. Sensitivity analysis: alternative population projection scenarios 13

Supplementary Table 6. Sensitivity analysis: income projection uncertainty (95% CIs for industrial workers’ wages and self-employed contribution points) 13

Supplementary Table 7. Sensitivity analysis: medical expense per capita projection uncertainty (95% CIs) 14

Supplementary Table 8. Sensitivity analysis: technology-diffusion and healthy aging scenario 14

Supplementary Table 9. Sensitivity analysis: contribution rate growth scenarios 15

Supplementary Table 1. Stationarity test: Augmented Dickey-Fuller (ADF) test statistics

| Variable | Tau Statistic  (Trend, lag=1) | p-value  (Trend, lag=1) | Stationarity (Yes/No) |
| --- | --- | --- | --- |
| Income (15-19) | -2.28 | 0.4206 | No |
| Income (20-24) | 0.00 | 0.9923 | No |
| Income (25-29) | -0.02 | 0.9919 | No |
| Income (30-34) | 0.52 | 0.9982 | No |
| Income (35-39) | 0.29 | 0.9966 | No |
| Income (40-44) | -0.05 | 0.9912 | No |
| Income (45-49) | -0.57 | 0.9668 | No |
| Income (50-54) | -1.36 | 0.8349 | No |
| Income (55-59) | -1.21 | 0.8743 | No |
| Income (60-64) | -1.28 | 0.8557 | No |
| Income (65-69) | -1.81 | 0.6567 | No |
| Income (70-74) | -2.08 | 0.5207 | No |
| Income (75+) | -2.20 | 0.3229 | No |
| Exp (0-4, survivors) | -3.19 | 0.1274 | No |
| Exp (5-9, survivors) | -3.83 | 0.0494 | Yes |
| Exp (10-14 survivors10) | -1.53 | 0.7603 | No |
| Exp (15-19, survivors) | -0.92 | 0.9204 | No |
| Exp (20-24, survivors) | 0.13 | 0.9929 | No |
| Exp (25-29 survivors) | -0.06 | 0.9885 | No |
| Exp (30-34, survivors) | -0.95 | 0.9161 | No |
| Exp (35-39, survivors) | -0.39 | 0.9748 | No |
| Exp (40-44, survivors) | -0.42 | 0.9728 | No |
| Exp (45-49, survivors) | 0.21 | 0.9943 | No |
| Exp (50-54, survivors) | -0.52 | 0.966 | No |
| Exp (55-59, survivors) | -1.98 | 0.5598 | No |
| Exp (60-64, survivors) | -2.19 | 0.455 | No |
| Exp (65-69, survivors) | -1.94 | 0.5807 | No |
| Exp (70-74, survivors) | -2.23 | 0.4376 | No |
| Exp (75-79, survivors) | -2.67 | 0.2616 | No |
| Exp (80-84, survivors) | -2.6 | 0.2853 | No |
| Exp (85-89, survivors) | -2.00 | 0.5482 | No |
| Exp (90+, survivors) | -1.98 | 0.5580 | No |
| Exp (0-4, decedents) | -2.42 | 0.3515 | No |
| Exp (5-9, decedents) | -1.59 | 0.7403 | No |
| Exp (10-14, decedents) | -3.12 | 0.1418 | No |
| Exp (15-19, decedents) | -2.02 | 0.5409 | No |
| Exp (20-24, decedents) | -3.45 | 0.0876 | No |
| Exp (25-29, decedents) | -2.38 | 0.3712 | No |
| Exp (30-34 decedents) | -2.3 | 0.4035 | No |
| Exp (35-39, decedents) | -2.64 | 0.2728 | No |
| Exp (40-44, decedents) | -2.3 | 0.4039 | No |
| Exp (45-49, decedents) | -2.29 | 0.4099 | No |
| Exp (50-54, decedents) | -2.11 | 0.497 | No |
| Exp (55-59, decedents) | -1.98 | 0.5598 | No |
| Exp (60-64, decedents) | -2.19 | 0.455 | No |
| Exp (65-69, decedents) | -1.94 | 0.5807 | No |
| Exp (70-74, decedents) | -2.23 | 0.4376 | No |
| Exp (75-79, decedents) | -1.77 | 0.6591 | No |
| Exp (80-84, decedents) | -2.08 | 0.5119 | No |
| Exp (85-89, decedents) | -2.31 | 0.4009 | No |
| Exp (90+, decedents) | -2.28 | 0.4122 | No |

Note:

1. Variables: ‘Income’ refers to average monthly income subject to NHI contribution for workplace subscribers. ‘Exp’ represents mean medical expense per capita. Numbers in parentheses indicate age groups. Survivors and decedents refer to those who survived and those who died during each period, respectively.

2. The Augmented Dickey-Fuller (ADF) test null hypothesis is that the time series has a unit root (non-stationary). Rejection of the null (p < 0.05) indicates the series is stationary.

Supplementary Table 2. ARIMAX model fit comparison (BIC) and model selection: income

| Exogenous variables | GDP per Capita | | | | Nominal Wage per Capita | | | |
| --- | --- | --- | --- | --- | --- | --- | --- | --- |
| Variable | ARIMAX (1,1,1) | ARIMAX (0,1,1) | ARIMAX (1,1,0) | ARIMAX (0,1,0) | ARIMAX (1,1,1) | ARIMAX (0,1,1) | ARIMAX (1,1,0) | ARIMAX (0,1,0) |
| Income (15-19)^1)^ | 464.1 | 459.3 | 462.0 | 459.5 | 440.5 | 434.2 | 436.9 | 434.6 |
| Income (20-24)^1)^ | 453.6 | 447.8 | 448.2 | 446.0 | 426.0 | 420.6 | 423.3 | 421.7 |
| Income (25-29) | 444.2 | 443.4 | 443.4 | 440.6 | 420.3 | 418.5 | 418.5 | 415.6 |
| Income (30-35) | 445.4 | 440.9 | 441.1 | 438.9 | 420.4 | 416.6 | 416.8 | 414.7 |
| Income (35-39) | 455.3 | 452.4 | 452.4 | 449.5 | 429.3 | 426.7 | 426.8 | 424.2 |
| Income (40-44)^1)^ | 463.1 | 460.3 | 460.3 | 457.4 | 435.5 | 428.1 | 432.5 | 429.8 |
| Income (45-49)^1)^ | 464.8 | 458.4 | 463.6 | 461.5 | 442.7 | 432.5 | 437.5 | 435.8 |
| Income (50-54) | 472.4 | 470.9 | 471.1 | 468.6 | 447.0 | 445.4 | 445.5 | 443.3 |
| Income (55-59)^1)^ | 464.5 | 461.9 | 464.5 | 462.6 | 442.0 | 439.8 | 440.2 | 438.3 |
| Income (60-64) | 461.2 | 459.4 | 459.4 | 456.6 | 465.9 | 434.3 | 434.5 | 431.9 |
| Income (65-69) | 456.4 | 450.5 | 454.9 | 452.1 | 429.7 | 429.1 | 429.4 | 426.8 |
| Income (70-74) | 467.3 | 465.0 | 467.1 | 464.5 | 442.0 | 435.9 | 436.0 | 433.2 |
| Income (75+) | 453.0 | 450.2 | 450.2 | 447.6 | 453.0 | 450.2 | 450.2 | 447.6 |

Note:

1. Light gray shading indicates variables exhibiting excessive volatility or non-systematic temporal variation unsuitable for ARIMAX modeling; projections for these variables were based on historical averages

2. Given that the null hypothesis of a unit root (non-stationarity) was not rejected based on the ADF test (supplementary Table 1), only ARIMAX models with differencing are considered from model selection

3. Model selection was based on three criteria: (1) stationarity (ADF) test, (2) BIC values, and (3) model stability and convergence. The shaded model indicates the final selection

4. The trend of model selection based on AIC was consistent with BIC across variables

1) ARIMAX (0,1,1) was excluded despite lowest BIC due to convergence issues; ARIMAX (0,1,0) was adopted based on BIC and model stability

Supplementary Table 3. ARIMAX model fit comparison (BIC) and model selection: expenditure of survivors

| Variable | Impact of COVID-19 | ARIMAX (1,1,1) | ARIMAX (0,1,1) | ARIMAX (1,1,0) | ARIMAX (0,1,1) |
| --- | --- | --- | --- | --- | --- |
| Exp (0-4, survivors) | 2020 | 365.1 | 363.3 | 363.3 | 360.7 |
|  | 2021 | 391.3 | 383.9 | 363.3 | 382.6 |
|  | 2022 | 384.5 | 379.0 | 382.2 | 380.3 |
|  | 2020-2021 | 380.8 | 373.3 | 373.3 | 370.7 |
|  | 2021-2022 | 385.7 | 383.6 | 373.3 | 381.9 |
|  | 2020-2022 | 388.1 | 379.7 | 373.3 | 379.5 |
|  | No impact | 385.7 | 382.5 | 382.7 | 380.1 |
| Exp (5-9, survivors) | 2020 | 381.4 | 358.1 | 382.6 | 356.9 |
|  | 2021 | 372.8 | 370.1 | 371.1 | 368.5 |
|  | 2022 | 371.5 | 370.8 | 371.1 | 368.5 |
|  | 2020-2021 | 357.4 | 370.8 | 355.4 | 353.8 |
|  | 2021-2022 | 373.2 | 370.9 | 355.4 | 369.9 |
|  | 2020-2022 | 365.1 | 363.1 | 365.1 | 364.1 |
|  | No impact | 370.7 | 368.4 | 369.2 | 367.2 |
| Exp (10-14, survivors) | 2020 | 338.3 | 335.7 | 335.8 | 335.2 |
|  | 2021 | 360.2 | 351.6 | 335.8 | 352.2 |
|  | 2022 | 345.5 | 343.3 | 345.1 | 346.8 |
|  | 2020-2021 | 346.0 | 344.6 | 345.1 | 342.7 |
|  | 2021-2022 | 354.1 | 349.5 | 345.1 | 350.0 |
|  | 2020-2022 | 357.5 | 351.3 | 353.3 | 351.1 |
|  | No impact | 354.1 | 348.4 | 351.9 | 349.7 |
| Exp (15-19 survivors) | 2020 | 332.7 | 330.2 | 330.1 | 332.4 |
|  | 2021 | 354.2 | 340.7 | 343.5 | 341.4 |
|  | 2022 | 327.9 | 325.4 | 327.3 | 328.0 |
|  | 2020-2021 | 344.3 | 337.3 | 327.3 | 335.6 |
|  | 2021-2022 | 352.5 | 337.4 | 327.3 | 336.1 |
|  | 2020-2022 | 344.7 | 340.3 | 343.5 | 341.4 |
|  | No impact | 347.4 | 337.5 | 340.9 | 338.8 |
| Exp (20-24, survivors)^1)^ | 2020 | 308.7 | 310.6 | 311.0 | 316.6 |
|  | 2021 | 315.9 | 309.6 | 311.0 | 317.6 |
|  | 2022 | 308.1 | 306.4 | 306.4 | 303.8 |
|  | 2020-2021 | 321.7 | 313.1 | 313.8 | 316.8 |
|  | 2021-2022 | 308.6 | 308.2 | 311.5 | 311.7 |
|  | 2020-2022 | 321.0 | 309.1 | 314.6 | 316.3 |
|  | No impact | 319.8 | 308.2 | 312.0 | 315.0 |
| Exp (25-29, survivors)^1)^ | 2020 | 317.3 | 315.4 | 316.0 | 320.7 |
|  | 2021 | 326.1 | 312.9 | 315.6 | 321.0 |
|  | 2022 | 314.0 | 312.5 | 312.6 | 310.1 |
|  | 2020-2021 | 317.3 | 314.0 | 317.3 | 321.0 |
|  | 2021-2022 | 320.3 | 312.1 | 312.7 | 313.4 |
|  | 2020-2022 | 321.1 | 311.8 | 316.4 | 318.0 |
|  | No impact | 320.8 | 311.0 | 314.7 | 318.4 |
| Exp (30-34 survivors) | 2020 | 322.7 | 319.2 | 323.0 | 322.7 |
|  | 2021 | 324.8 | 321.2 | 323.0 | 324.9 |
|  | 2022 | 324.6 | 319.7 | 323.0 | 319.8 |
|  | 2020-2021 | 329.2 | 327.4 | 323.0 | 325.0 |
|  | 2021-2022 | 321.0 | 318.4 | 320.6 | 318.6 |
|  | 2020-2022 | 328.3 | 320.1 | 325.9 | 324.7 |
|  | No impact | 327.1 | 317.4 | 323.3 | 322.8 |
| Exp (35-39, survivors)^2)^ | 2020 | 321.0 | 317.3 | 320.0 | 318.5 |
|  | 2021 | 330.9 | 322.7 | 320.0 | 327.1 |
|  | 2022 | 327.5 | 319.4 | 324.2 | 322.8 |
|  | 2020-2021 | 328.4 | 320.6 | 324.2 | 324.4 |
|  | 2021-2022 | 325.8 | 324.8 | 325.5 | 322.9 |
|  | 2020-2022 | 330.9 | 322.2 | 328.8 | 327.3 |
|  | No impact | 328.2 | 319.6 | 326.3 | 324.7 |
| Exp (40-44, survivors)^2)^ | 2020 | 318.8 | 315.1 | 318.2 | 317.2 |
|  | 2021 | 330.7 | 322.6 | 318.2 | 327.0 |
|  | 2022 | 328.4 | 320.7 | 325.3 | 323.8 |
|  | 2020-2021 | 327.9 | 318.0 | 323.7 | 322.7 |
|  | 2021-2022 | 328.1 | 321.9 | 327.2 | 324.7 |
|  | 2020-2022 | 330.5 | 321.1 | 327.6 | 326.5 |
|  | No impact | 327.9 | 319.4 | 326.0 | 324.4 |
| Exp (45-49, survivors) | 2020 | 325.6 | 323.0 | 323.0 | 320.4 |
|  | 2021 | 330.4 | 324.9 | 328.8 | 326.6 |
|  | 2022 | 330.7 | 323.1 | 326.8 | 325.6 |
|  | 2020-2021 | 329.7 | 324.5 | 326.6 | 324.5 |
|  | 2021-2022 | 329.4 | 323.7 | 327.7 | 325.3 |
|  | 2020-2022 | 330.7 | 325.0 | 328.4 | 326.4 |
|  | No impact | 327.4 | 322.3 | 326.1 | 324.2 |
| Exp (50-54, survivors) | 2020 | 331.7 | 329.5 | 329.5 | 326.9 |
|  | 2021 | 341.1 | 333.0 | 337.4 | 335.4 |
|  | 2022 | 339.3 | 333.5 | 337.5 | 335.4 |
|  | 2020-2021 | 334.0 | 330.7 | 333.2 | 331.8 |
|  | 2021-2022 | 340.1 | 333.2 | 337.5 | 335.4 |
|  | 2020-2022 | 336.6 | 331.3 | 334.4 | 332.3 |
|  | No impact | 336.4 | 330.9 | 334.9 | 332.8 |
| Exp (55-59, survivors) | 2020 | 345.6 | 343.4 | 343.5 | 340.9 |
|  | 2021 | 348.3 | 345.0 | 347.6 | 345.6 |
|  | 2022 | 350.9 | 344.9 | 347.5 | 344.9 |
|  | 2020-2021 | 347.2 | 344.6 | 344.6 | 342.7 |
|  | 2021-2022 | 346.9 | 342.9 | 347.0 | 345.1 |
|  | 2020-2022 | 343.7 | 340.8 | 342.4 | 340.2 |
|  | No impact | 347.6 | 345.3 | 345.4 | 343.0 |
| Exp (60-64, survivors) | 2020 | 353.8 | 351.7 | 351.7 | 349.2 |
|  | 2021 | 359.1 | 353.6 | 356.2 | 353.9 |
|  | 2022 | 357.4 | 354.1 | 356.3 | 353.8 |
|  | 2020-2021 | 355.5 | 353.2 | 353.3 | 351.0 |
|  | 2021-2022 | 358.7 | 352.8 | 356.1 | 353.8 |
|  | 2020-2022 | 356.2 | 352.8 | 352.8 | 350.4 |
|  | No impact | 355.5 | 353.7 | 353.8 | 351.3 |
| Exp (65-69, survivors)^3)^ | 2020 | 367.3 | 365.1 | 365.2 | 363.7 |
|  | 2021 | 372.3 | 371.0 | 371.0 | 368.3 |
|  | 2022 | 371.3 | 370.5 | 370.6 | 368.0 |
|  | 2020-2021 | 369.6 | 367.2 | 367.2 | 364.6 |
|  | 2021-2022 | 372.9 | 370.3 | 370.3 | 367.7 |
|  | 2020-2022 | 366.3 | 364.9 | 364.9 | 362.3 |
|  | No impact | 370.4 | 368.3 | 368.4 | 365.8 |
| Exp (70-74, survivors)^4)^ | 2020 | 379.0 | 371.7 | 371.7 | 369.1 |
|  | 2021 | 377.9 | 374.1 | 375.8 | 374.4 |
|  | 2022 | 382.1 | 375.8 | 376.1 | 373.7 |
|  | 2020-2021 | 376.7 | 372.0 | 372.1 | 370.7 |
|  | 2021-2022 | 378.1 | 370.5 | 375.0 | 373.7 |
|  | 2020-2022 | 371.4 | 364.4 | 368.8 | 367.6 |
|  | No impact | 378.3 | 374.1 | 374.2 | 371.8 |
| Exp (75-79, survivors)^5)^ | 2020 | 376.3 | 373.6 | 369.7 | 371.0 |
|  | 2021 | 380.5 | 372.3 | 374.4 | 376.1 |
|  | 2022 | 380.1 | 376.2 | 378.3 | 375.7 |
|  | 2020-2021 | 375.2 | 368.7 | 370.0 | 369.9 |
|  | 2021-2022 | 380.4 | 369.4 | 375.1 | 374.2 |
|  | 2020-2022 | 368.4 | 358.0 | 362.6 | 362.6 |
|  | No impact | 378.6 | 376.7 | 376.8 | 374.2 |
| Exp (80-84, survivors)^4)^ | 2020 | 376.9 | 374.8 | 370.2 | 372.2 |
|  | 2021 | 371.8 | 372.7 | 373.8 | 377.2 |
|  | 2022 | 382.1 | 378.3 | 380.3 | 377.7 |
|  | 2020-2021 | 368.2 | 364.8 | 367.3 | 368.8 |
|  | 2021-2022 | 379.9 | 372.8 | 376.9 | 375.1 |
|  | 2020-2022 | 359.5 | 355.4 | 359.7 | 359.4 |
|  | No impact | 381.2 | 378.7 | 378.7 | 376.1 |
| Exp (85-89, survivors)^5)^ | 2020 | 379.8 | 377.4 | 372.2 | 374.9 |
|  | 2021 | 377.1 | 375.6 | 377.7 | 378.2 |
|  | 2022 | 382.3 | 379.8 | 381.9 | 380.5 |
|  | 2020-2021 | 397.2 | 364.0 | 365.0 | 367.3 |
|  | 2021-2022 | 399.8 | 378.9 | 378.9 | 376.3 |
|  | 2020-2022 | 359.1 | 352.7 | 353.5 | 354.9 |
|  | No impact | 381.7 | 379.8 | 380.4 | 378.6 |
| Exp (90+, survivors)^5)^ | 2020 | 378.3 | 375.8 | 377.3 | 376.9 |
|  | 2021 | 383.3 | 380.9 | 382.0 | 379.4 |
|  | 2022 | 380.3 | 377.7 | 381.4 | 381.2 |
|  | 2020-2021 | 375.2 | 372.6 | 372.6 | 370.0 |
|  | 2021-2022 | 380.8 | 378.8 | 379.0 | 376.5 |
|  | 2020-2022 | 354.1 | 352.2 | 353.5 | 354.5 |
|  | No impact | 381.8 | 379.2 | 379.9 | 379.7 |

Note:

1. Given that the null hypothesis of a unit root (non-stationarity) was not rejected based on the ADF test (supplementary Table 1), only ARIMAX models with differencing are considered from model selection

2. Model selection was based on three criteria: (1) stationarity (ADF) test, (2) BIC values, and (3) model stability and convergence. The shaded model indicates the final selection

3. The trend of model selection based on AIC was consistent with BIC across variables

1) ARIMAX (0,1,1) with COVID (2022) was excluded despite lowest BIC due to convergence issues; ARIMAX (0,1,0) with COVID (2022) was adopted based on BIC and model stability

2) ARIMAX (0,1,1) with COVID (2020) was excluded despite lowest BIC due to convergence issues; ARIMAX (0,1,0) with COVID (2020) was adopted based on BIC and model stability

3) ARIMAX (0,1,0) with COVID (2020) was excluded despite lowest BIC due to convergence issues; ARIMAX (0,1,1) with COVID (2020) was adopted based on BIC and model stability

4) ARIMAX (0,1,1) with COVID (2020-2022) was excluded despite lowest BIC due to convergence issues; ARIMAX (0,1,0) with COVID (2020-2022) was adopted based on BIC and model stability

5) ARIMAX (0,1,1) with COVID (2020-2022) was excluded despite lowest BIC due to convergence issues; ARIMAX (1,1,0) with COVID (2020-2022) was adopted based on BIC and model stability

Supplementary Table 4. ARIMAX model fit comparison (BIC) and model selection: expenditure of decedents

| Variable | Impact of COVID-19 | ARIMAX (1,1,1) | ARIMAX (0,1,1) | ARIMAX (1,1,0) | ARIMAX (0,1,0) |
| --- | --- | --- | --- | --- | --- |
| Exp (0-4, decedents) | 2020 | NA | 480.6 | 481.1 | 479.3 |
|  | 2021 | 481.2 | 473.8 | 475.0 | 478.1 |
|  | 2022 | 472.6 | 469.4 | 472.1 | 469.8 |
|  | 2020-2021 | 480.6 | 476.7 | 479.5 | 478.7 |
|  | 2021-2022 | 469.0 | **460.5** | 462.2 | 465.3 |
|  | 2020-2022 | 474.6 | 466.1 | 471.0 | 470.7 |
|  | No impact | NA | 478.0 | 478.5 | 476.6 |
| Exp (5-9, decedents) | 2020 | 473.2 | 471.4 | 471.7 | 469.7 |
|  | 2021 | 480.0 | 478.0 | 481.6 | 480.9 |
|  | 2022 | 483.7 | 479.9 | 481.6 | 480.3 |
|  | 2020-2021 | 479.5 | 472.3 | 475.3 | 475.9 |
|  | 2021-2022 | 483.6 | **478.1** | 482.1 | 480.4 |
|  | 2020-2022 | 481.0 | 471.5 | 477.9 | 478.7 |
|  | No impact | 481.1 | 477.6 | 479.5 | 478.3 |
| Exp (10-14, decedents)^1)^ | 2020 | 487.4 | 482.8 | 485.2 | 484.8 |
|  | 2021 | 484.7 | 477.1 | 480.8 | 484.2 |
|  | 2022 | 484.6 | 477.0 | 481.3 | 483.1 |
|  | 2020-2021 | 489.9 | 480.9 | 481.3 | 486.7 |
|  | 2021-2022 | 473.9 | **473.2** | 474.9 | 476.7 |
|  | 2020-2022 | 486.8 | 478.4 | 482.4 | 484.9 |
|  | No impact | 487.6 | 478.5 | 483.3 | 484.1 |
| Exp (15-19 decedents)^1)^ | 2020 | 466.4 | **464.5** | 468.8 | 471.1 |
|  | 2021 | 473.0 | 473.3 | 470.7 | 476.2 |
|  | 2022 | 474.7 | 473.6 | 473.3 | 476.3 |
|  | 2020-2021 | 464.7 | 465.5 | 466.3 | 472.1 |
|  | 2021-2022 | 474.4 | 474.6 | 473.8 | 476.3 |
|  | 2020-2022 | 470.2 | 469.7 | 469.8 | 472.7 |
|  | No impact | 474.1 | 475.4 | 471.6 | 473.7 |
| Exp (20-24, decedents) | 2020 | 443.1 | 441.0 | 443.8 | 445.2 |
|  | 2021 | 445.5 | 444.2 | 445.7 | 448.8 |
|  | 2022 | 442.1 | 441.4 | 445.3 | 448.3 |
|  | 2020-2021 | 442.7 | 441.8 | 444.8 | 451.0 |
|  | 2021-2022 | 442.9 | 442.2 | 445.5 | 451.4 |
|  | 2020-2022 | 443.6 | 442.5 | 444.1 | 448.2 |
|  | 2020, 2022 | 440.2 | 437.1 | 439.3 | 436.8 |
|  | No impact | 440.2 | 439.5 | 443.1 | 448.7 |
| Exp (25-29, decedents)^1)^ | 2020 | 436.4 | 434.9 | 435.5 | 433.0 |
|  | 2021 | 439.5 | 433.7 | 437.7 | 437.2 |
|  | 2022 | 442.8 | 440.5 | 440.5 | 437.9 |
|  | 2020-2021 | 432.9 | 426.5 | 430.8 | 430.6 |
|  | 2021-2022 | 438.0 | 435.4 | 438.4 | 436.1 |
|  | 2020-2022 | 428.5 | 421.9 | 427.9 | 426.6 |
|  | No impact | 440.6 | 438.4 | 438.4 | 435.8 |
| Exp (30-34 decedents)^1)^ | 2020 | 423.0 | 437.2 | 423.7 | 426.8 |
|  | 2021 | 425.8 | 422.1 | 424.9 | 429.7 |
|  | 2022 | 427.1 | 435.3 | 426.5 | 429.8 |
|  | 2020-2021 | 422.0 | 432.4 | 422.6 | 427.5 |
|  | 2021-2022 | 429.1 | 422.9 | 426.5 | 429.8 |
|  | 2020-2022 | 426.2 | 426.5 | 424.0 | 428.1 |
|  | No impact | 424.2 | 432.4 | 424.2 | 427.2 |
| Exp (35-39, decedents)^2)^ | 2020 | 432.0 | 424.6 | 429.4 | 427.8 |
|  | 2021 | 429.2 | 427.2 | 428.3 | 432.3 |
|  | 2022 | 436.9 | 430.6 | 432.9 | 431.5 |
|  | 2020-2021 | 426.9 | 423.9 | 426.5 | 428.5 |
|  | 2021-2022 | 431.6 | 428.5 | 430.8 | 431.2 |
|  | 2020-2022 | 427.4 | 422.9 | 425.9 | 424.7 |
|  | No impact | 432.5 | 427.6 | 430.4 | 429.8 |
| Exp (40-44, decedents)^1)^ | 2020 | 438.6 | 437.1 | 437.0 | 434.8 |
|  | 2021 | 438.0 | 440.9 | 442.2 | 445.9 |
|  | 2022 | 457.6 | 441.8 | 445.0 | 445.4 |
|  | 2020-2021 | 438.5 | 437.7 | 436.4 | 441.6 |
|  | 2021-2022 | 449.3 | 439.9 | 443.1 | 445.8 |
|  | 2020-2022 | 430.9 | 429.7 | 433.4 | 438.9 |
|  | No impact | 443.4 | 441.2 | 442.6 | 443.3 |
| Exp (45-49, decedents)^3)^ | 2020 | 431.5 | 428.9 | 428.8 | 427.2 |
|  | 2021 | 430.8 | 429.1 | 429.0 | 427.2 |
|  | 2022 | 424.3 | 420.0 | 423.6 | 423.1 |
|  | 2020-2021 | 430.5 | 429.0 | 428.9 | 427.5 |
|  | 2021-2022 | 432.8 | 422.1 | 425.9 | 426.4 |
|  | 2020-2022 | 427.3 | 424.3 | 426.2 | 425.5 |
|  | No impact | 427.8 | 426.5 | 426.3 | 424.9 |
| Exp (50-54, decedents)^1)^ | 2020 | 433.3 | 431.1 | 431.2 | 428.8 |
|  | 2021 | 434.0 | 425.7 | 426.7 | 428.0 |
|  | 2022 | 429.1 | 424.4 | 426.7 | 424.1 |
|  | 2020-2021 | 434.2 | 428.7 | 428.9 | 427.6 |
|  | 2021-2022 | 427.3 | 415.7 | 420.3 | 420.9 |
|  | 2020-2022 | 425.1 | 417.7 | 420.5 | 420.2 |
|  | No impact | 435.0 | 428.9 | 428.9 | 426.5 |
| Exp (55-60, decedents)^1)^ | 2020 | 439.3 | 437.2 | 437.3 | 436.2 |
|  | 2021 | 436.7 | 435.0 | 436.1 | 438.1 |
|  | 2022 | 437.4 | 430.5 | 433.6 | 431.2 |
|  | 2020-2021 | 438.2 | 434.5 | 435.6 | 437.1 |
|  | 2021-2022 | 437.1 | 428.9 | 431.9 | 434.4 |
|  | 2020-2022 | 428.5 | 425.6 | 427.6 | 429.4 |
|  | No impact | 438.9 | 436.4 | 436.3 | 435.4 |
| Exp (60-64, decedents)^4)^ | 2020 | 442.2 | 438.1 | 438.3 | 436.6 |
|  | 2021 | 439.9 | 433.4 | 434.5 | 436.5 |
|  | 2022 | 433.2 | 428.3 | 431.3 | 428.8 |
|  | 2020-2021 | 441.9 | 435.3 | 436.7 | 436.2 |
|  | 2021-2022 | 434.2 | 423.6 | 426.6 | 429.2 |
|  | 2020-2022 | 433.0 | 425.2 | 429.0 | 429.2 |
|  | No impact | 439.8 | 435.8 | 435.9 | 434.2 |
| Exp (65-69, decedents)^4)^ | 2020 | 460.3 | 441.1 | 442.4 | 440.8 |
|  | 2021 | 439.6 | 435.0 | 435.9 | 440.1 |
|  | 2022 | 439.7 | 433.8 | 437.0 | 434.5 |
|  | 2020-2021 | 444.2 | 437.2 | 439.6 | 439.9 |
|  | 2021-2022 | 435.9 | 428.4 | 429.8 | 432.0 |
|  | 2020-2022 | 435.4 | 428.0 | 431.0 | 432.3 |
|  | No impact | 459.6 | 439.6 | 440.1 | 438.4 |
| Exp (70-74, decedents)^4)^ | 2020 | 441.4 | 439.5 | 439.6 | 437.3 |
|  | 2021 | 438.9 | 433.7 | 434.2 | 436.5 |
|  | 2022 | 433.4 | 432.0 | 434.2 | 430.1 |
|  | 2020-2021 | 439.8 | 436.1 | 437.0 | 436.2 |
|  | 2021-2022 | 429.2 | 422.2 | 426.0 | 426.9 |
|  | 2020-2022 | 428.3 | 423.1 | 426.3 | 426.7 |
|  | No impact | 439.6 | 437.3 | 437.3 | 434.9 |
| Exp (75-79, decedents)^4)^ | 2020 | 434.9 | 431.4 | 433.2 | 431.9 |
|  | 2021 | 424.1 | 426.8 | 423.9 | 431.8 |
|  | 2022 | 427.7 | 423.8 | 427.2 | 424.6 |
|  | 2020-2021 | 430.2 | 427.0 | 429.5 | 431.0 |
|  | 2021-2022 | 416.1 | 418.0 | 418.0 | 423.3 |
|  | 2020-2022 | 425.7 | 416.4 | 418.8 | 420.6 |
|  | No impact | 432.1 | 429.7 | 431.2 | 429.8 |
| Exp (80-84, decedents)^4)^ | 2020 | 427.9 | 426.5 | 426.8 | 425.7 |
|  | 2021 | 423.9 | 421.6 | 421.3 | 426.0 |
|  | 2022 | 418.9 | 414.3 | 418.0 | 415.4 |
|  | 2020-2021 | 427.0 | 423.5 | 424.9 | 425.5 |
|  | 2021-2022 | 406.7 | 411.9 | 410.5 | 418.0 |
|  | 2020-2022 | 419.3 | 413.6 | 416.1 | 416.5 |
|  | No impact | 425.3 | 424.3 | 424.4 | 423.5 |
| Exp (85-89, decedents)^4)^ | 2020 | 427.4 | 424.0 | 424.3 | 423.4 |
|  | 2021 | 419.3 | 419.0 | 416.7 | 423.6 |
|  | 2022 | 421.1 | 412.1 | 416.7 | 412.4 |
|  | 2020-2021 | 424.4 | 420.2 | 421.8 | 423.0 |
|  | 2021-2022 | 399.0 | 405.4 | 402.0 | 414.2 |
|  | 2020-2022 | 411.9 | 407.8 | 410.9 | 412.6 |
|  | No impact | 424.2 | 421.9 | 421.9 | 421.2 |
| Exp (90+, decedents)^4)^ | 2020 | 418.9 | 415.3 | 416.8 | 416.2 |
|  | 2021 | 411.4 | 411.1 | 408.8 | 416.2 |
|  | 2022 | 407.8 | 402.9 | 406.5 | 404.1 |
|  | 2020-2021 | 418.0 | 412.8 | 415.0 | 415.9 |
|  | 2021-2022 | 385.8 | 397.5 | 389.6 | 406.4 |
|  | 2020-2022 | 407.2 | 403.2 | 406.2 | 406.1 |
|  | No impact | 417.1 | 412.8 | 414.1 | 413.9 |

Note: NA, not applicable

1. Given that the null hypothesis of a unit root (non-stationarity) was not rejected based on the ADF test (supplementary Table 1), only ARIMAX models with differencing are considered from model selection

2. Model selection was based on three criteria: (1) stationarity (ADF) test, (2) BIC values, and (3) model stability and convergence. The shaded model indicates the final selection

3. The trend of model selection based on AIC was consistent with BIC across variables

1) ARIMAX (0,1,1) with COVID (2020-2022) was excluded despite lowest BIC due to convergence issues; ARIMAX (1,1,0) with COVID (2020-2022) was adopted based on BIC and model stability

2) ARIMAX (0,1,1) with COVID (2020-2022) was excluded despite lowest BIC due to convergence issues; ARIMAX (0,1,0) with COVID (2020-2022) was adopted based on BIC and model stability

3) ARIMAX (0,1,1) with COVID (2022) was excluded despite lowest BIC due to convergence issues; ARIMAX (0,1,0) with COVID (2022) was adopted based on BIC and model stability

4) ARIMAX (0,1,1) with COVID (2021-2022) was excluded despite lowest BIC due to convergence issues; ARIMAX (1,1,0) with COVID (2021-2022) was adopted based on BIC and model stability

Supplementary Table 5. Sensitivity analysis: alternative population projection scenarios

unit: trillion KRW

|  |  | 2023 | 2032 | 2042 | Average annual growth rate (2023-2032) | Average annual growth rate (2033-2042) |
| --- | --- | --- | --- | --- | --- | --- |
| Medium scenario  (baseline) | NHI revenue | 94.9 | 181.8 | 314.2 | 7.5% | 7.7% |
|  | NHI expenditure | 90.8 | 203.7 | 437.5 | 9.4% | 9.6% |
|  | Fiscal balance | 4.1 | -21.8 | -123.3 |  |  |
|  | Accumulated reserves | 28.0 | -46.9 | -781.9 |  |  |
| High scenario | NHI revenue | 94.9 | 184.9 | 330.8 | 7.7% | 7.9% |
|  | NHI expenditure | 90.8 | 207.0 | 454.4 | 9.6% | 9.8% |
|  | Fiscal balance | 4.1 | -22.0 | -123.6 |  |  |
|  | Accumulated reserves | 28.0 | -49.7 | -787.1 |  |  |
| Low scenario | NHI revenue | 94.9 | 177.4 | 296.7 | 7.2% | 7.3% |
|  | NHI expenditure | 90.8 | 200.2 | 420.0 | 9.2% | 9.4% |
|  | Fiscal balance | 4.1 | -22.8 | -123.3 |  |  |
|  | Accumulated reserves | 28.0 | -52.7 | -794.9 |  |  |

**Note: NHI, National Health Insurance;** **1USD = 1,382 KRW **(****as of July 31, 2025****)****

1. Population projection scenarios are based on Statistics Korean official projections: medium (baseline), high scenario (high fertility, low mortality, high net immigration), and low scenario (low fertility, high mortality, low net immigration)

Supplementary Table 6. Sensitivity analysis: income projection uncertainty (95% CIs for industrial workers’ wages and self-employed contribution points)

unit: trillion KRW

|  |  | 2023 | 2032 | 2042 | Average annual growth rate (2023-2032) | Average annual growth rate (2033-2042) |
| --- | --- | --- | --- | --- | --- | --- |
| Point estimate  (baseline) | NHI revenue | 94.9 | 181.8 | 314.2 | 7.5% | 5.6% |
|  | NHI expenditure | 90.8 | 203.7 | 437.5 | 9.4% | 7.8% |
|  | Fiscal balance | 4.1 | -21.8 | -123.3 |  |  |
|  | Accumulated reserves | 28.0 | -46.9 | -781.9 |  |  |
| Upper  95% CI | NHI revenue | 94.9 | 191.8 | 328.0 | 8.1% | 5.5% |
|  | NHI expenditure | 90.8 | 203.7 | 437.5 | 9.4% | 7.8% |
|  | Fiscal balance | 4.1 | -11.8 | -109.5 |  |  |
|  | Accumulated reserves | 28.0 | 11.0 | -604.3 |  |  |
| Lower  95% CI | NHI revenue | 94.9 | 170.9 | 298.4 | 6.8% | 5.7% |
|  | NHI expenditure | 90.8 | 203.7 | 437.5 | 9.4% | 7.8% |
|  | Fiscal balance | 4.1 | -32.8 | -139.1 |  |  |
|  | Accumulated reserves | 28.0 | -112.2 | -985.4 |  |  |

**Note:** 95% CIs, 95% confidence intervals; **NHI, National Health Insurance;** **1USD = 1,382 KRW **(****as of July 31, 2025****)****

1. 95% CIs were derived from ARIMAX model prediction intervals for applicable variables; for variables unsuitable for ARIMAX modeling, a ±3% range was applied to point estimates

Supplementary Table 7. Sensitivity analysis: medical expense per capita projection uncertainty (95% CIs)

unit: trillion KRW

|  |  | 2023 | 2032 | 2042 | Average annual growth rate (2023-2032) | Average annual growth rate (2033-2042) |
| --- | --- | --- | --- | --- | --- | --- |
| Point estimate  (baseline) | NHI revenue | 94.9 | 181.8 | 314.2 | 7.5% | 5.6% |
|  | NHI expenditure | 90.8 | 203.7 | 437.5 | 9.4% | 7.8% |
|  | Fiscal balance | 4.1 | -21.8 | -123.3 |  |  |
|  | Accumulated reserves | 28.0 | -46.9 | -781.9 |  |  |
| Upper  95% CI | NHI revenue | 94.9 | 181.8 | 314.2 | 7.5% | 5.6% |
|  | NHI expenditure | 90.8 | 213.2 | 452.5 | 9.9% | 7.7% |
|  | Fiscal balance | 4.1 | -31.4 | -138.3 |  |  |
|  | Accumulated reserves | 28.0 | -104.2 | -966.2 |  |  |
| Lower  95% CI | NHI revenue | 94.9 | 182.4 | 314.2 | 7.5% | 5.6% |
|  | NHI expenditure | 90.8 | 194.5 | 424.5 | 8.8% | 8.0% |
|  | Fiscal balance | 4.1 | -12.2 | -110.3 |  |  |
|  | Accumulated reserves | 28.0 | 9.8 | -609.6 |  |  |

**Note:** 95% CIs, 95% confidence intervals; **NHI, National Health Insurance;** **1USD = 1,382 KRW **(****as of July 31, 2025****)****

1. 95% CIs were derived from ARIMAX model prediction intervals for applicable variables; for variables unsuitable for ARIMAX modeling, a ±3% range was applied to point estimates

Supplementary Table 8. Sensitivity analysis: technology-diffusion and healthy aging scenarios

unit: trillion KRW

|  |  | 2023 | 2032 | 2042 | Average annual growth rate (2023-2032) | Average annual growth rate (2033-2042) |
| --- | --- | --- | --- | --- | --- | --- |
| baseline | NHI revenue | 94.9 | 181.8 | 314.2 | 7.5% | 5.6% |
|  | NHI expenditure | 90.8 | 203.7 | 437.5 | 9.4% | 7.8% |
|  | Fiscal balance | 4.1 | -21.8 | -123.3 |  |  |
|  | Accumulated reserves | 28.0 | -46.9 | -781.9 |  |  |
| 3% lower expenses (65+) | NHI revenue | 94.9 | 181.8 | 314.2 | 7.5% | 5.6% |
|  | NHI expenditure | 90.8 | 200.2 | 428.9 | 9.2% | 7.8% |
|  | Fiscal balance | 4.1 | -18.4 | -114.7 |  |  |
|  | Accumulated reserves | 28.0 | -26.6 | -700.7 |  |  |
| 5% lower expenses (65+) | NHI revenue | 94.9 | 181.9 | 314.2 | 7.5% | 5.6% |
|  | NHI expenditure | 90.8 | 197.9 | 423.1 | 9.0% | 7.8% |
|  | Fiscal balance | 4.1 | -16.0 | -108.9 |  |  |
|  | Accumulated reserves | 28.0 | -12.9 | -646.4 |  |  |

**Note: NHI, National Health Insurance;** **1USD = 1,382 KRW **(****as of July 31, 2025****)****

1. Scenarios assume annual per capita medical expenses for those aged 65 and older are reduced by 3% and 5% relative to baseline levels (97% and 95% of baseline, respectively), reflecting potential cost moderation from accelerated healthy aging or digital health innovations (e.g., AI, telemedicine).

Supplementary Table 9. Sensitivity analysis: contribution rate growth scenarios

unit: trillion KRW

|  |  | 2023 | 2032 | 2042 | Average annual growth rate (2023-2032) | Average annual growth rate (2033-2042) |
| --- | --- | --- | --- | --- | --- | --- |
| 1.48%,  no ceiling  (baseline) | NHI revenue | 94.9 | 181.8 | 314.2 | 7.5% | 5.6% |
|  | NHI expenditure | 90.8 | 203.7 | 437.5 | 9.4% | 7.8% |
|  | Fiscal balance | 4.1 | -21.8 | -123.3 |  |  |
|  | Accumulated reserves | 28.0 | -46.9 | -781.9 |  |  |
| 1.48%,  8% ceiling | NHI revenue | 94.9 | 181.8 | 275.8 | 7.5% | 4.1% |
|  | NHI expenditure | 90.8 | 203.7 | 437.5 | 9.4% | 7.8% |
|  | Fiscal balance | 4.1 | -21.8 | -161.7 |  |  |
|  | Accumulated reserves | 28.0 | -46.9 | -952.0 |  |  |
| 1%,  no ceiling | NHI revenue | 94.9 | 176.6 | 290.9 | 7.1% | 5.1% |
|  | NHI expenditure | 90.8 | 203.7 | 437.5 | 9.4% | 7.8% |
|  | Fiscal balance | 4.1 | -27.1 | -146.6 |  |  |
|  | Accumulated reserves | 28.0 | -64.1 | -937.8 |  |  |
| 1%,  8% ceiling | NHI revenue | 94.9 | 176.6 | 275.8 | 7.1% | 4.5% |
|  | NHI expenditure | 90.8 | 203.7 | 437.5 | 9.4% | 7.8% |
|  | Fiscal balance | 4.1 | -27.1 | -161.7 |  |  |
|  | Accumulated reserves | 28.0 | -64.1 | -984.6 |  |  |
| 3%,  no ceiling | NHI revenue | 94.9 | 199.0 | 398.5 | 8.6% | 7.2% |
|  | NHI expenditure | 90.8 | 203.7 | 437.5 | 9.4% | 7.8% |
|  | Fiscal balance | 4.1 | -4.7 | -39.0 |  |  |
|  | Accumulated reserves | 28.0 | 6.0 | -242.8 |  |  |
| 1.48%,  8% ceiling | NHI revenue | 94.9 | 184.2 | 274.7 | 7.6% | 4.1% |
|  | NHI expenditure | 90.8 | 203.7 | 437.5 | 9.4% | 7.8% |
|  | Fiscal balance | 4.1 | -19.5 | -162.8 |  |  |
|  | Accumulated reserves | 28.0 | -20.8 | -934.1 |  |  |

**Note:** 95% CIs, 95% confidence intervals; **NHI, National Health Insurance**

1. Scenarios combine annual contribution rate growth of 1%, 1.48% (baseline), and 3% per year, with or without application of the 8% statutory ceiling on the contribution rate itself
