# Supplementary material for: The bill of aging: fiscal projections of demographic changes on South Korea’s national health insurance, 2023–2042
Source: Health Econ Rev. 2025 Nov 17;15:97. doi: 10.1186/s13561-025-00690-z (PMC12625416; doi:10.1186/s13561-025-00690-z)
Supplement: Supplementary file 2 — Supplementary Material 2. [file 13561_2025_690_MOESM2_ESM.docx]

**Supplementary Materials 2**

**List of Supplementary Figures**

Supplementary Figure 1. Trends in Korean National Health Insurance finances (2011-2022) 2

Supplementary Figure 2. Trends in NHI contributors: industrial workers and self-employed individuals 3

Supplementary Figure 3. Medical expenses by age group: survivors and decedents, 2023-2042 4

Supplementary Figure 4. Average medical expense per capita, 2023-2042 5

Supplementary Figure 5. Sensitivity analysis: alternative population projection scenarios 6

Supplementary Figure 6. Sensitivity analysis: income projection uncertainty (95% CIs for industrial workers’ wages and self-employed contribution points) 7

Supplementary Figure 7. Sensitivity analysis: medical expense per capita projection uncertainty (95% CIs) 8

Supplementary Figure 8. Sensitivity analysis: technology-diffusion and healthy aging scenario 9

Supplementary Figure 9. Sensitivity analysis: contribution rate growth scenarios 10

Supplementary Figure 10. Break-even contribution rate simulation for fiscal balance 11


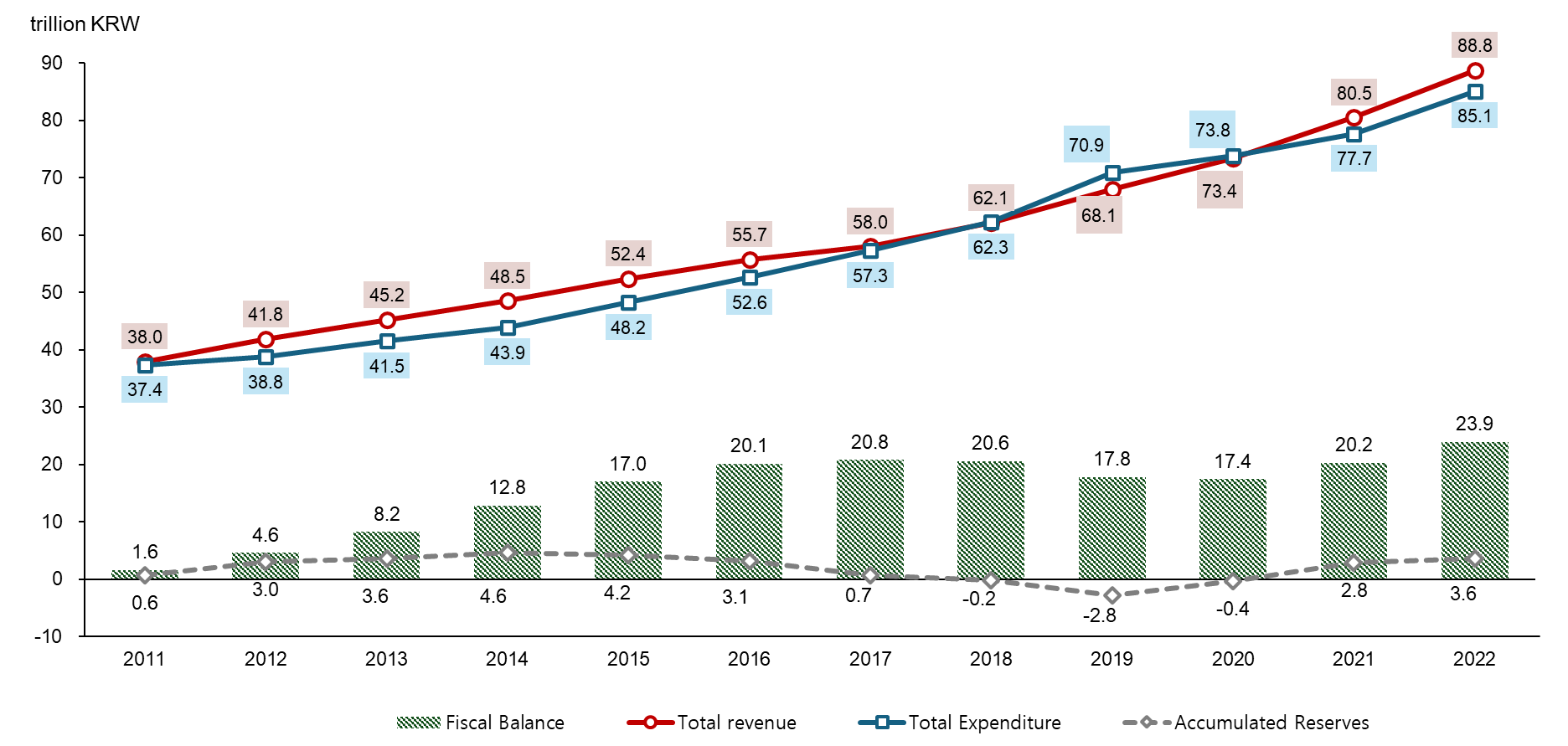


Supplementary Figure 1. Trends in Korean National Health Insurance finances (2011-2022)

Note: KRW, Korean Won; **1USD = 1,382 KRW (**as of July 31, 2025**)**


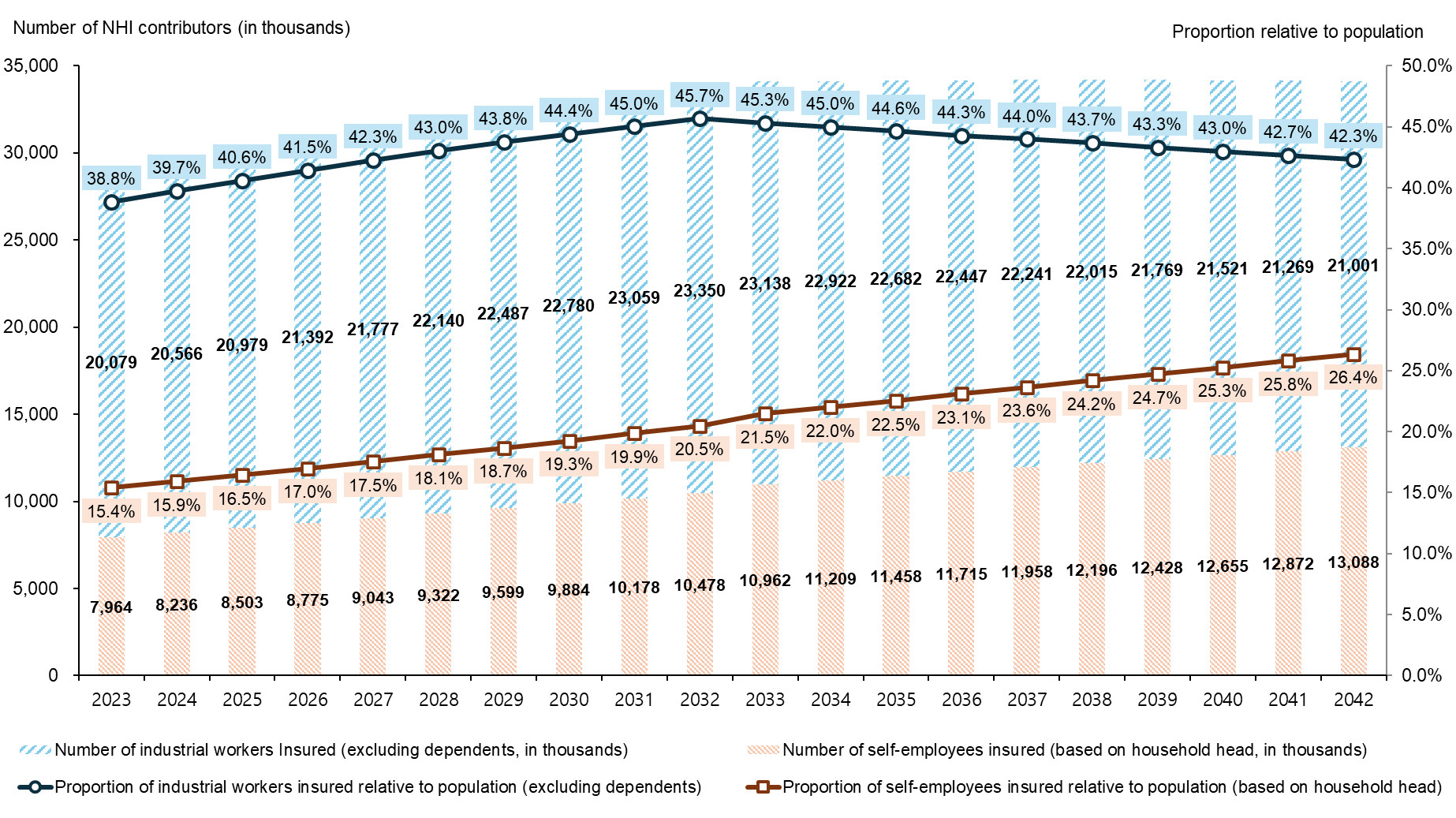


Supplementary Figure 2. Trends in NHI contributors: industrial workers and self-employed individuals


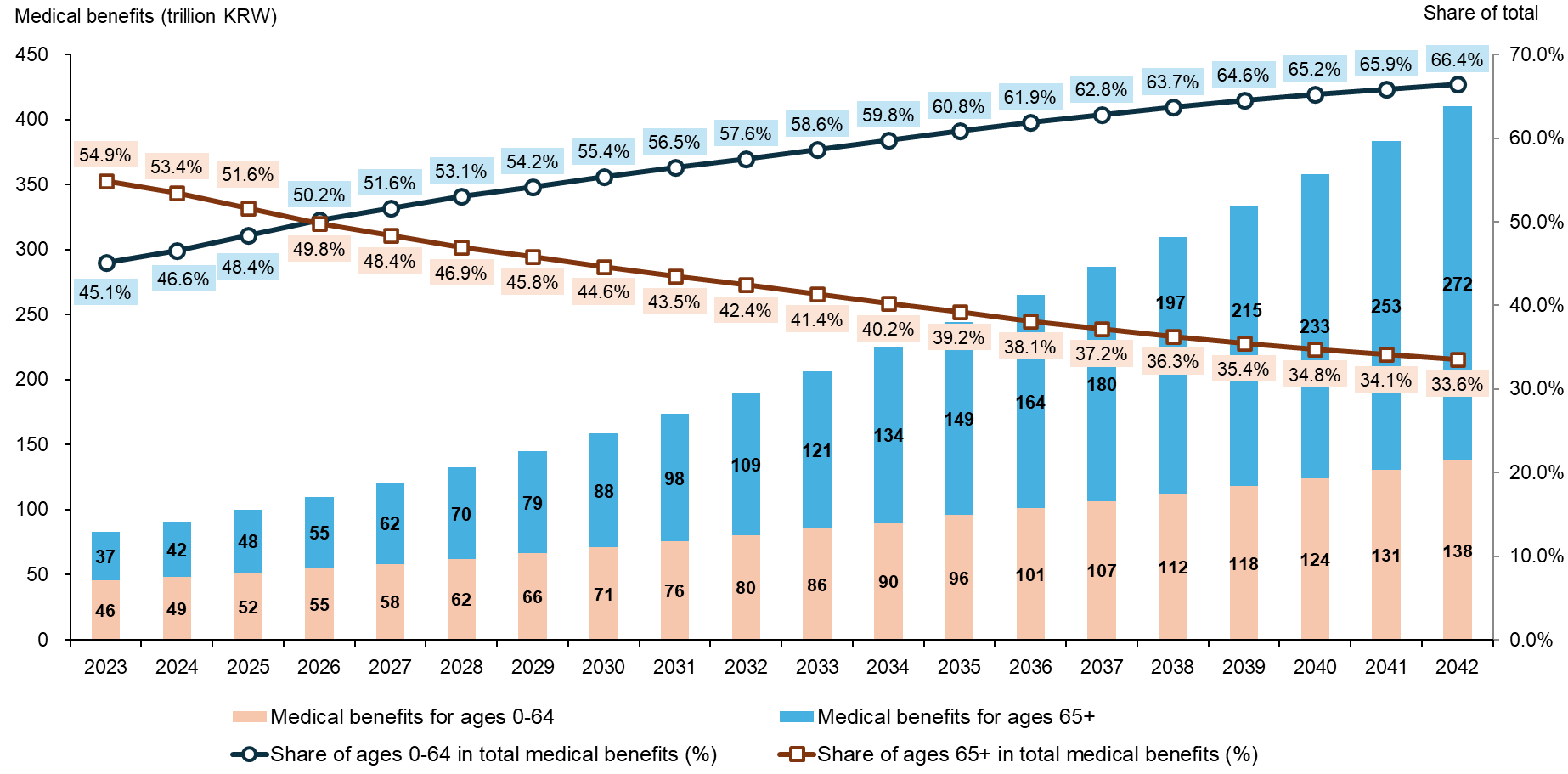


Supplementary Figure 3. Medical expenses by age group: survivors and decedents, 2023-2042

Note: KRW, Korean Won; **1USD = 1,382 KRW (**as of July 31, 2025**)**


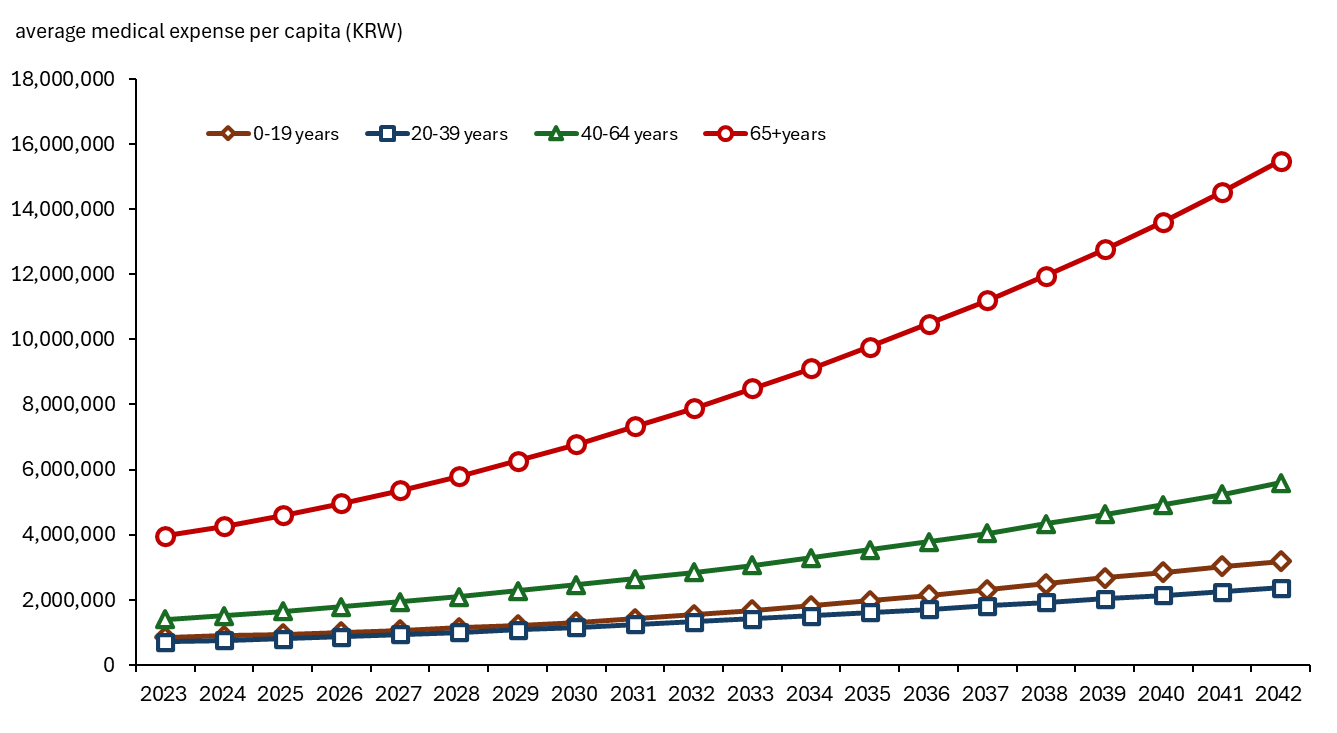


Supplementary Figure 4. Average medical expense per capita, 2023-2042

Note: KRW, Korean Won; **1USD = 1,382 KRW (**as of July 31, 2025**);** Average medical expense per capita for each age group is calculated by summing the total medical expenses for survivors and decedents (each group’s per capita expense multiplied by their respective number) and dividing by the total population in that age group.


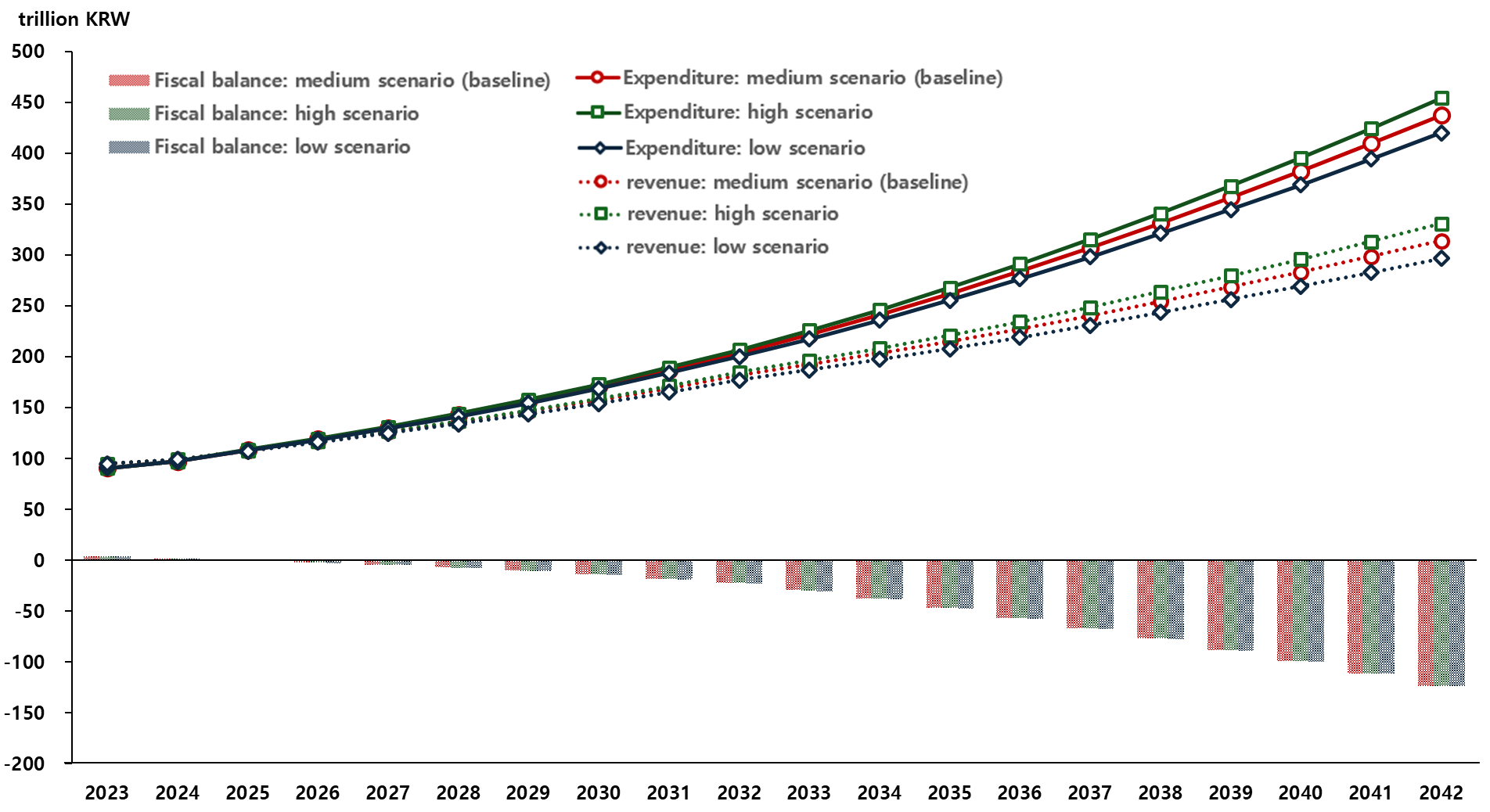


Supplementary Figure 5. Sensitivity analysis: alternative population projection scenarios

Note: KRW, Korean Won; 1USD = 1,382 KRW (as of July 31, 2025)

1. Population projection scenarios are based on Statistics Korean official projections: medium (baseline), high scenario (high fertility, low mortality, high net immigration), and low scenario (low fertility, high mortality, low net immigration)


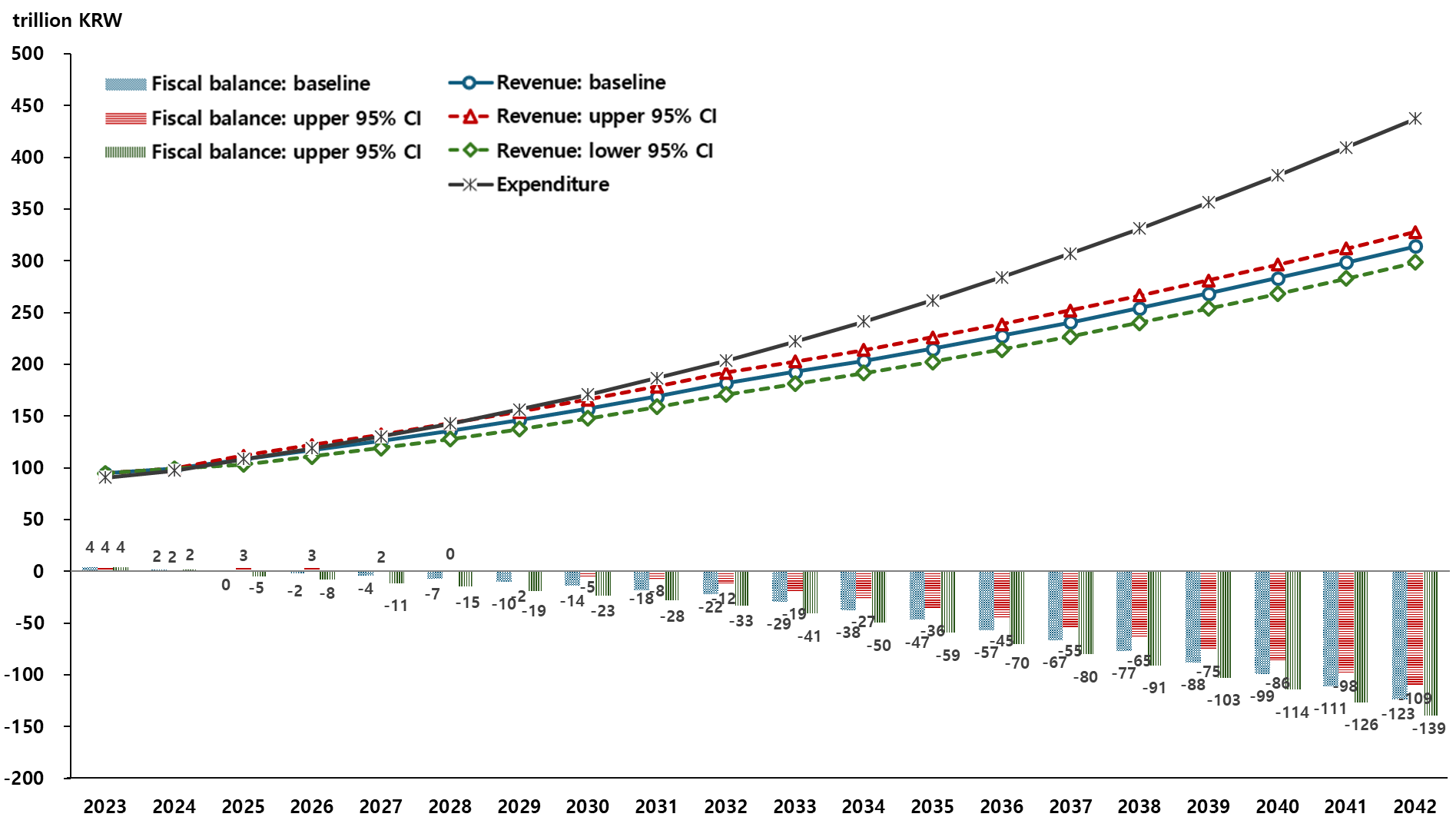


Supplementary Figure 6. Sensitivity analysis: income projection uncertainty (industrial workers’ wages and self-employed contribution points)

Note: KRW, Korean Won; 1USD = 1,382 KRW (as of July 31, 2025)

1. 95% CIs were derived from ARIMAX model prediction intervals for applicable variables; for variables unsuitable for ARIMAX modeling, a ±3% range was applied to point estimates


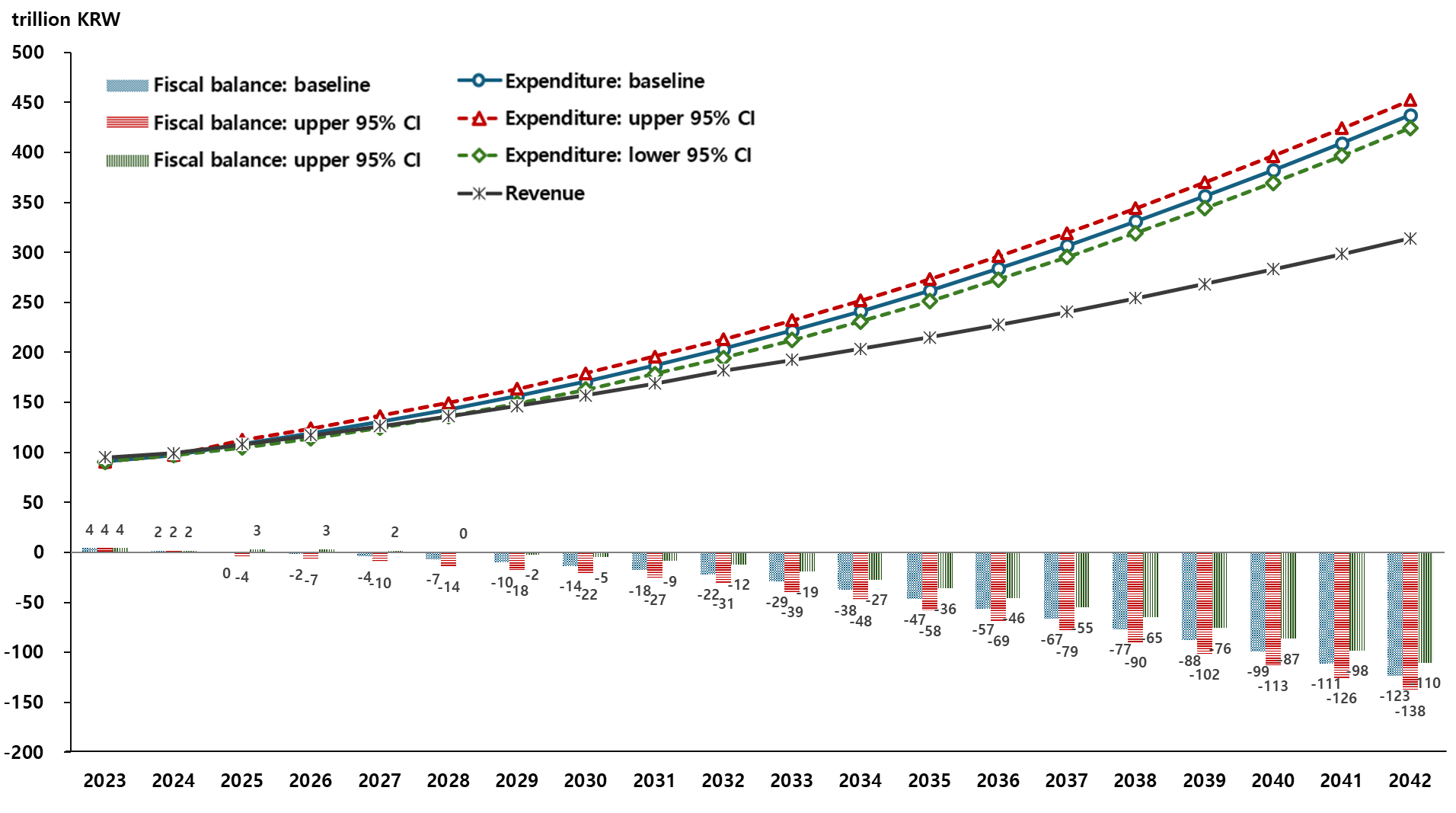


Supplementary Figure 7. Sensitivity analysis: medical expense per capita projection uncertainty

Note: KRW, Korean Won; **1USD = 1,382 KRW (**as of July 31, 2025**)**

**1.** 95% CIs were derived from ARIMAX model prediction intervals for applicable variables; for variables unsuitable for ARIMAX modeling, a ±3% range was applied to point estimates


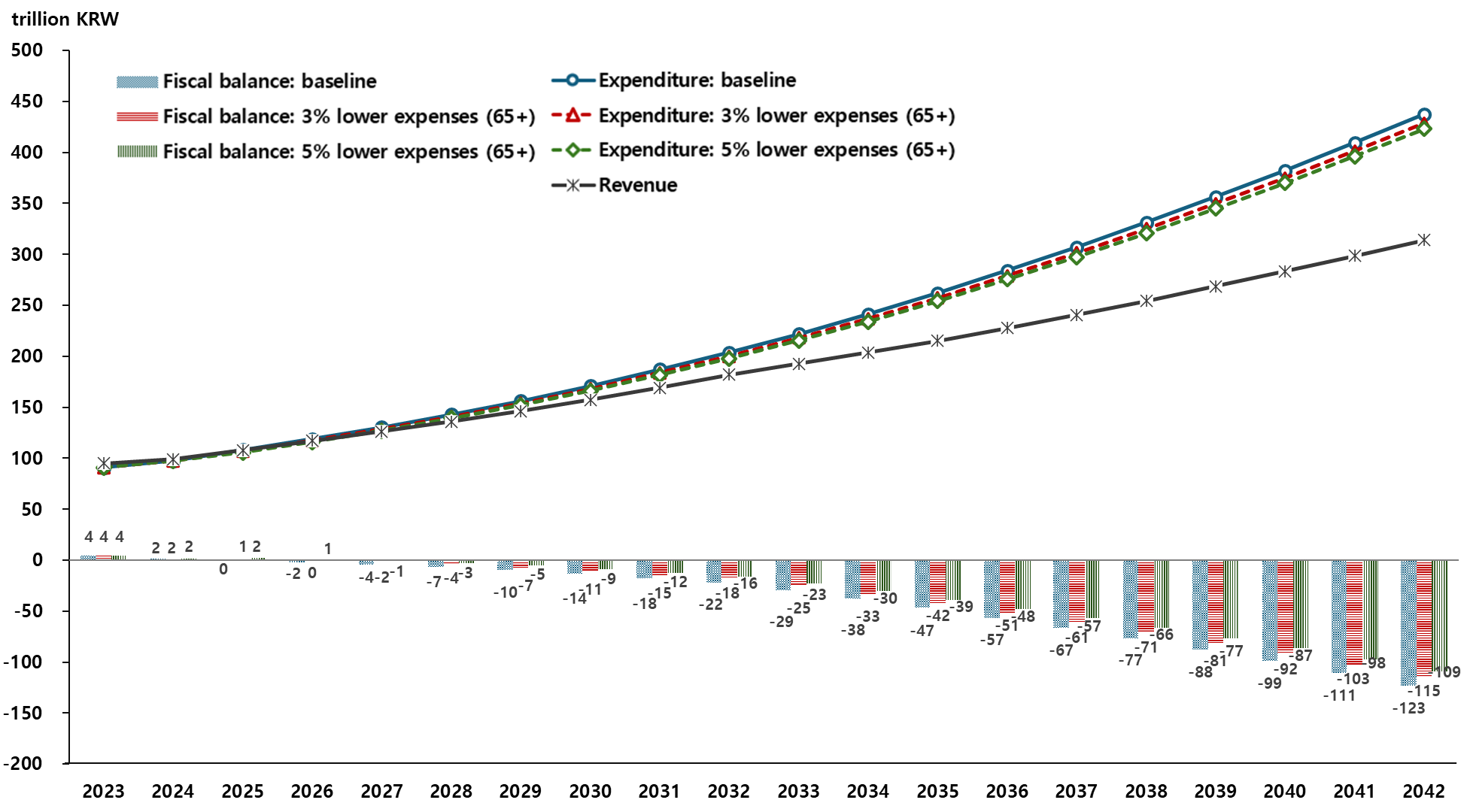


Supplementary Figure 8. Sensitivity analysis: technology-diffusion and healthy aging scenarios

Note: KRW, Korean Won; 1USD = 1,382 KRW (as of July 31, 2025)

1. Scenarios assume annual per capita medical expenses for those aged 65 and older are reduced by 3% and 5% relative to baseline levels (97% and 95% of baseline, respectively), reflecting potential cost moderation from accelerated healthy aging or digital health innovations (e.g., AI, telemedicine).


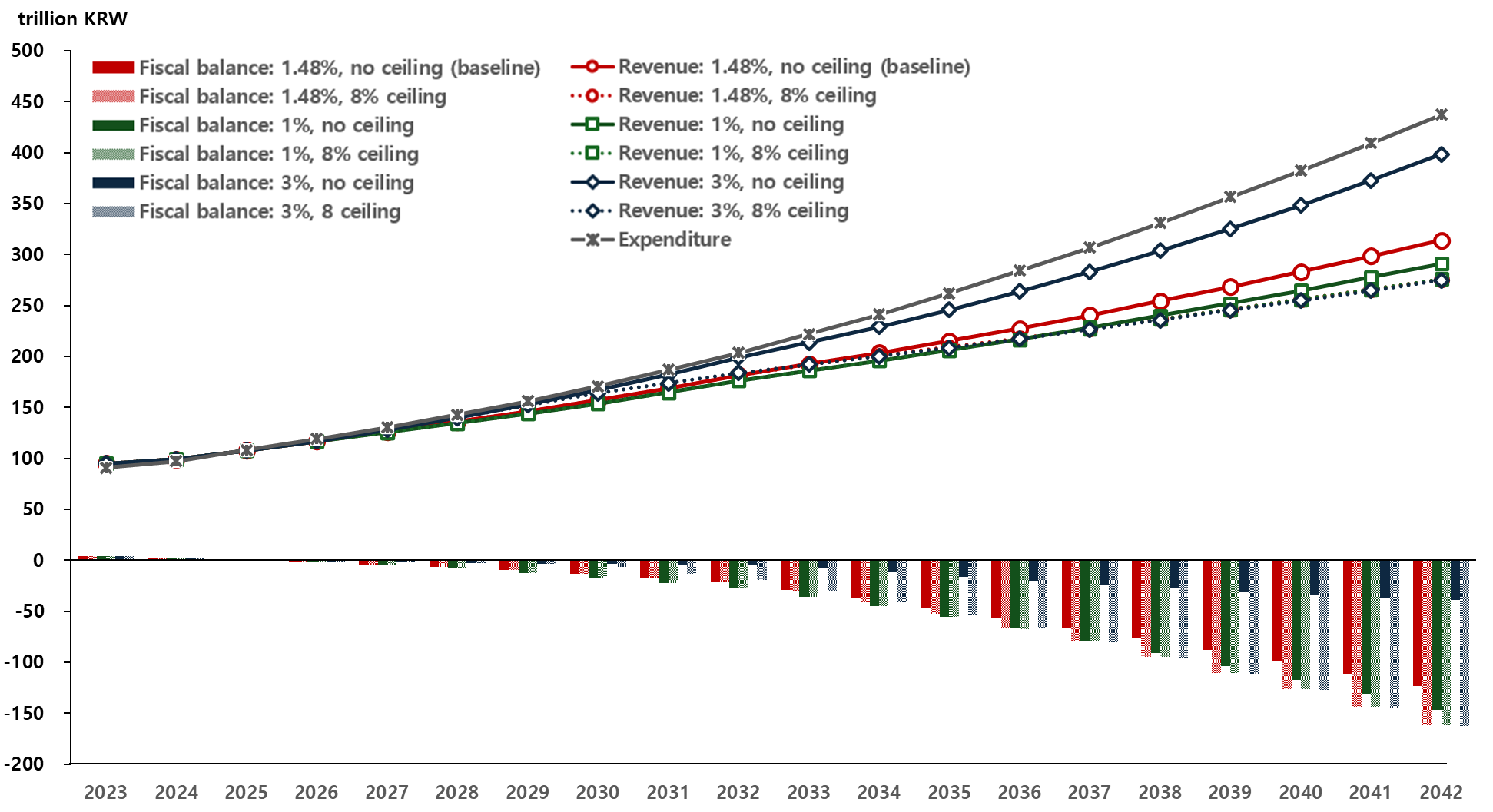


Supplementary Figure 9. Sensitivity analysis: contribution rate growth scenarios

Note: KRW, Korean Won; 1USD = 1,382 KRW (as of July 31, 2025)

1. Scenarios combine annual contribution rate growth of 1%, 1.48% (baseline), and 3% per year, with or without application of the 8% statutory ceiling on the contribution rate itself


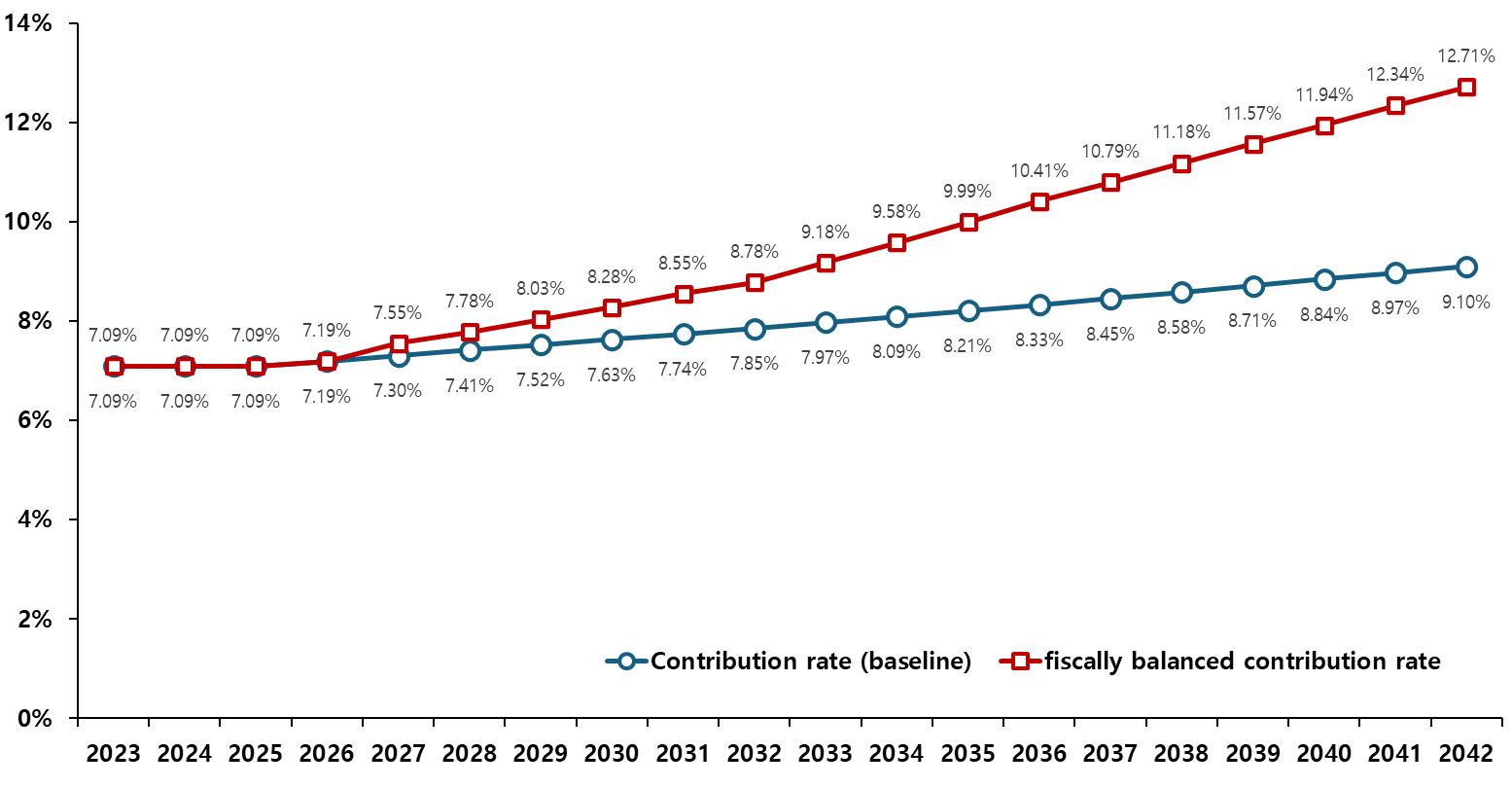


Supplementary Figure 10. Break-even contribution rate simulation for fiscal balance

Note: Starting from 2027 (as 2026 rates are predetermined), minimum contribution rates required to ensure annual revenues equal or exceed expenditures were iteratively calculated to prevent fiscal deficits
